# CRISPR-Cas9 genome editing induces megabase-scale chromosomal truncations

**DOI:** 10.1038/s41467-019-09006-2

**Published:** 2019-03-08

**Authors:** Grégoire Cullot, Julian Boutin, Jérôme Toutain, Florence Prat, Perrine Pennamen, Caroline Rooryck, Martin Teichmann, Emilie Rousseau, Isabelle Lamrissi-Garcia, Véronique Guyonnet-Duperat, Alice Bibeyran, Magalie Lalanne, Valérie Prouzet-Mauléon, Béatrice Turcq, Cécile Ged, Jean-Marc Blouin, Emmanuel Richard, Sandrine Dabernat, François Moreau-Gaudry, Aurélie Bedel

**Affiliations:** 1 Univ. Bordeaux, 33000 Bordeaux, France; 2 INSERM U1035, Biotherapy of genetic diseases, inflammatory disorders and cancers, 33000 Bordeaux, France; 3 Biochemistry Laboratory, CHU Bordeaux, 33000 Bordeaux, France; 4 Medical genetic laboratory, CHU Bordeaux, 33000 Bordeaux, France; 5 UMR 5320, INSERM U1212, ARNA Laboratory, 33000 Bordeaux, France; 6 Vectorology Platform, 33000 Bordeaux, France; 7 INSERM U1218, ACTION, 33000 Bordeaux, France; 8 Laboratory of excellence, GR-Ex, Imagine institute, 75015 Paris, France

## Abstract

CRISPR-Cas9 is a promising technology for genome editing. Here we use Cas9 nuclease-induced double-strand break DNA (DSB) at the *UROS* locus to model and correct congenital erythropoietic porphyria. We demonstrate that homology-directed repair is rare compared with NHEJ pathway leading to on-target indels and causing unwanted dysfunctional protein. Moreover, we describe unexpected chromosomal truncations resulting from only one Cas9 nuclease-induced DSB in cell lines and primary cells by a p53-dependent mechanism. Altogether, these side effects may limit the promising perspectives of the CRISPR-Cas9 nuclease system for disease modeling and gene therapy. We show that the single nickase approach could be safer since it prevents on- and off-target indels and chromosomal truncations. These results demonstrate that the single nickase and not the nuclease approach is preferable, not only for modeling disease but also and more importantly for the safe management of future CRISPR-Cas9-mediated gene therapies.

## Abstract (web summary)

CRISPR-Cas9 has been rapidly adopted to generate cell line models of disease. Here the authors show, while attempting to establish a congenital erythropoietic porphyria model, unexpected chromosome truncations generated by a p53-dependent mechanism.

## Introduction

CRISPR-Cas9 is an RNA-guided DNA endonuclease system targeting a specific genomic sequence complementary to a single-guide RNA (sgRNA) and juxtaposed with a protospacer adjacent motif (PAM). This system leads to a DNA double-strand break (DSB) via the RuvC and HNH nuclease domains of the Cas9 enzyme^1–4^. Most publications report the use of engineered Cas9-nucleases to efficiently induce DSBs at sites of interest^5–7^. DSBs lead to non-conservative non-homologous end-joining (NHEJ) repair pathway. Insertions or deletions (indels) at the on-target site often cause frameshifts in open reading frames and knockout (KO) genes. CRISPR-Cas9 applications are of particular interest to invalidate genes in the field of human genetics for disease modeling in vitro and in vivo^8^ and are promising for gene therapy. Sichuan University (China) was the first to submit a trial that consisted in injecting gene-edited cells in a person to evaluate the safety of *PD-1* knockout engineered T cells in treating metastatic non-small cell lung cancer^9^. A prospective phase 1 trial will start in the USA for patients with melanoma, synovial sarcoma, and multiple myeloma^10^. However, the CRISPR-Cas9 approach faces concerns regarding unintended alterations (off-target effect)^11^. Safety issues regarding genomic instability and chromosomal integrity have not been explored in-depth and could be underestimated. Indeed, CRISPR-Cas9 has already been applied to generate intra-chromosomal translocations to obtain fusion genes such as the *EML4-ALK* oncogene^12,13^ and inter/intra-chromosomal translocations in human HEK293T cells^14^. Recently, Adikusuma *et al*. reported frequent large deletions (kilobase-scale) in mouse zygotes after CRISPR-Cas9 cleavage^15^. Chromosomal deletions have been described in zebrafish using two DSBs by TALENs (Transcription activator-like effector nucleases) or CRISPR-Cas9 systems^16^. Recently, Zuo et al. surprisingly demonstrated that multiple Cas9-mediated DNA cleavages on the same human chromosome can eliminate it, thereby offering a new approach for modeling aneuploidy diseases^16–18^. However, although nuclease is the standard approach for genome editing, it is still unknown whether a part or an entire chromosome could be eliminated after only one DSB in human cells.

Another goal of using CRISPR-Cas9 is the possibility to perform homology-directed repair (HDR) to make precise genome editing (PGE) to insert or correct point mutations. This holds promise for correcting most hereditary diseases due to mutation hotspots. For example, congenital erythropoietic porphyria (CEP [MIM 263700]) is an autosomal recessive disorder characterized by a deficiency in the enzymatic activity of uroporphyrinogen III synthase (UROS; EC 4.2.1.75), the fourth enzyme of the heme biosynthetic pathway (Supplementary Figure 1). c.217 T > C substitution is a hotspot for this disease in almost one-third of all reported CEP cases^19–21^. The enzymatic defect causes the accumulation of the uroporphyrin I. The lack of UROS causes mutilating dermatological lesions resulting from the release of photocatalytic porphyrins and transfusion-dependent hemolytic anemia with secondary hypersplenism. The precise correction of mutation hotspots, without insertional mutagenesis and with physiological gene regulation, is the holy grail of gene therapy. However, performing HDR is still challenging due to low efficiency and concurrent misrepair of DSBs by the NHEJ. It is still unclear whether large amounts of unwanted on-target indels could ablate the residual function of the original protein, thereby modifying the phenotype of cells and leading to iatrogenic effects.

**Fig. 1:**
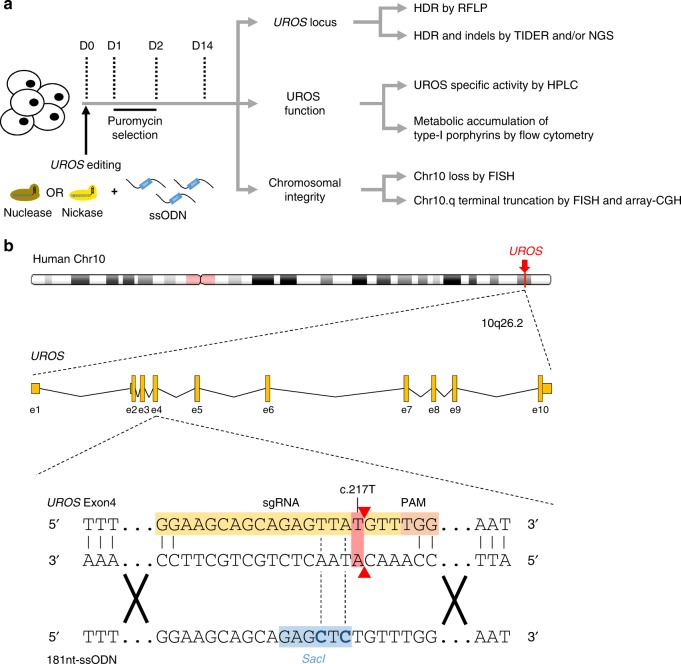
*UROS* gene editing strategy and workflow analysis. **a** Experimental workflow for *UROS* gene editing and analysis of outcomes. Cells were nucleofected with the 181nt-ssODN template and either with nuclease or nickase followed by puromycin-positive selection. Then, (i) *UROS* locus was characterized by RFLP to quantify HDR and by TIDER or deep sequencing to evaluate indels and to confirm HDR percentage; (ii) UROS functionality was assessed by quantifying UROS-specific activity and type-I porphyrin accumulation, respectively determined by HPLC and flow cytometry; (iii) Chromosomal integrity was tested for Chr10 loss or Chr10q terminal deletion either by DNA-FISH assay or array-CGH. **b** (Top) Schematic *UROS* locus in chromosome 10 with *UROS* gene overview (middle). (Bottom) Detailed view of exon 4 region and CRISPR-mediated HDR design using a c.217T-targeting sgRNA (highlighted in orange) with adjacent PAM and an 181nt-ssODN carrying a silent *SacI* restriction site (highlighted in blue) close to c.217 T position. Red arrows indicate expected cleavage site using nuclease. Chr chromosome, CGH comparative genomic hybridization, D day, e exon, HDR homology-directed repair, HPLC high performance liquid chromatography, NGS (next-generation sequencing), RFLP (restriction fragment length polymorphism), PAM protospacer adjacent motif, sgRNA single guide RNA, TIDER (tracking of insertions, deletions and recombination events)

Because error-prone NHEJ is the main repair pathway of DNA DSB, replacement of DSBs by single-strand break (SSB) could improve PGE by limiting indels. Nicks are the most common forms of DNA damage and elegant proof-of-principle has been provided that single nickase can initiate HDR^22,23^. Even if nuclease is still the gold standard system used to edit the genome^24^, some authors^25–27^ recently proposed that Cas9^D10A^-nickase could be an alternative to nuclease to reduce NHEJ. However, in this innovative approach, the HDR rate is often under 1%. Hence, knowing whether single nickase is potentially an alternative to nuclease is an area of active investigation. In this work, we characterize allelic outcomes and chromosomal integrity following either nuclease-induced DSB or nickase-induced SSB and monitored the HDR/indel ratio as a quality parameter of PGE (Fig. 1a). Using *UROS* as a target gene provide an easy and quantitative test of UROS function with detection of pathologic type-I porphyrins by flow cytometry. Our findings reveal the globally damaging effects of DSBs on the human genome in cell lines and primary cells in which the p53 tumor suppressor has been inactivated. They also highlight the possibility of using the single nickase approach to dramatically reduce indels and chromosomal terminal deletion while achieving a high HDR rate. This approach is therefore more relevant for testing disease models and for obtaining safer gene therapies.

## Results

### Profound KO is concurrent to knock-in using Cas9 nuclease

**Fig. 2:**
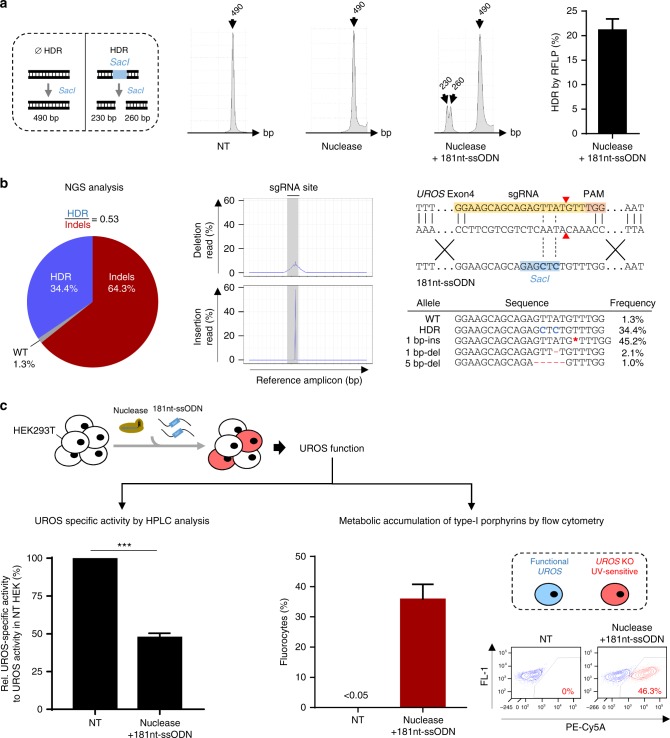
Nuclease-mediated HDR is associated with predominant indels leading to impaired UROS functionality. **a** (Left) Scheme of *SacI*-digested PCR products obtained for alleles with or without HDR. (Center) Illustrative RFLP analysis of non-transfected HEK293T cells (NT) or transfected with nuclease only or co-delivered with the 181nt-ssODN template. (Right) HDR frequency induced by nuclease and a 181nt-ssODN (*n* = 5). **b** (Left) NGS analysis of allelic outcomes and associated HDR/indel ratio following transfection of HEK293T cells with nuclease and 181nt-ssODN. (Center) Frequencies of reads carrying either insertion or deletion. Region spanning sgRNA sequence is highlighted in grey. (Right) Most common observed alleles (with frequencies ≥ 1%) aligned on the sgRNA sequence. HDR modification in blue and indels in red. **c** (Left) Quantification of UROS-specific activity from HEK293T cells NT or transfected with nuclease and 181nt-ssODN (*n* = 3). Values are normalized with NT cells. (Right) Fluorocyte frequencies and illustrative flow cytometry results from NT or HEK293T cells transfected with nuclease and a 181nt-ssODN (*n* = 5). Blue and red dots (and associated percentages) depict non-fluorescent cells and fluorocytes, respectively, with type-I porphyrin accumulation. Results are presented as mean ± SEM. The data are from independent experiments. Statistical significance is inferred on raw data using two-tailed unpaired *t* test for UROS-specific activity. ***p < 0.001. For **a**, **c**, source data are provided as a Source data file

*UROS* gene was edited in HEK293T cells by transient expression of Cas9-nuclease, a sgRNA and 181nt-single-stranded oligodeoxynucleotide (ssODN) template. To induce a DSB close to the most frequent mutation in CEP disease (c.217 T > C in *UROS* exon 4, chromosome 10), we designed a sgRNA inducing a DSB near the c.217 position and devised a 181nt-ssODN carrying a silent *SacI* restriction site close to the c.217 T position (Fig. 1b). Since HDR-mediated *SacI* insertion will lead to a modification of the sgRNA seed sequence, this event prevents Cas9-nuclease from re-cutting. We obtained a 21 ± 2.4% (*n* = 5) HDR rate by RLFP (restriction fragment length polymorphism) (Fig. 2a). TIDER (Tracking of Insertions, DEletions and Recombination events) analysis confirmed the presence of *SacI* (21.8% of alleles) and revealed a high rate of unwanted indels (60.7%) (Supplementary Figure 2a). Next-generation sequencing (NGS) analysis validated the HDR rate (34.4% of alleles) and high level of indels (Fig. 2b left panel). The predominant event is an insertion of one base pair at the cut site (Fig. 2b center and right panels). Only 1.3% of alleles were unmodified, attesting to the extremely high efficiency of Cas9-nuclease. To measure the impact of indels, we carried out an UROS enzymatic assay on transfected cells. This indicated a dramatic drop in UROS activity (47.6% ± 2.8% compared to non-transfected cells, *n* = 3) (Fig. 2c left panel). UROS dysfunction induced the striking appearance of a high level of fluorocytes (35.7% ± 5.1%, *n* = 3) (Fig. 2c right panel) due to accumulation of type-I porphyrins in cells. In parallel with on-target analysis, we evaluated the off-target effects of nuclease by quantifying indels in the top 10 off-target sequences predicted by CRISPOR. We observed indels in the first three ranked loci, located in intergenic regions, in accordance with the well-known low specificity of nuclease (Supplementary Table 1). Altogether, using the nuclease approach, we found the simultaneous presence of HDR with unacceptable on-target indels, leading to frequent KO of the *UROS* gene, to a metabolic deficiency, and to off-target side-effects.

### Cas9 nuclease induces unwanted chromosomal truncations

**Fig. 3:**
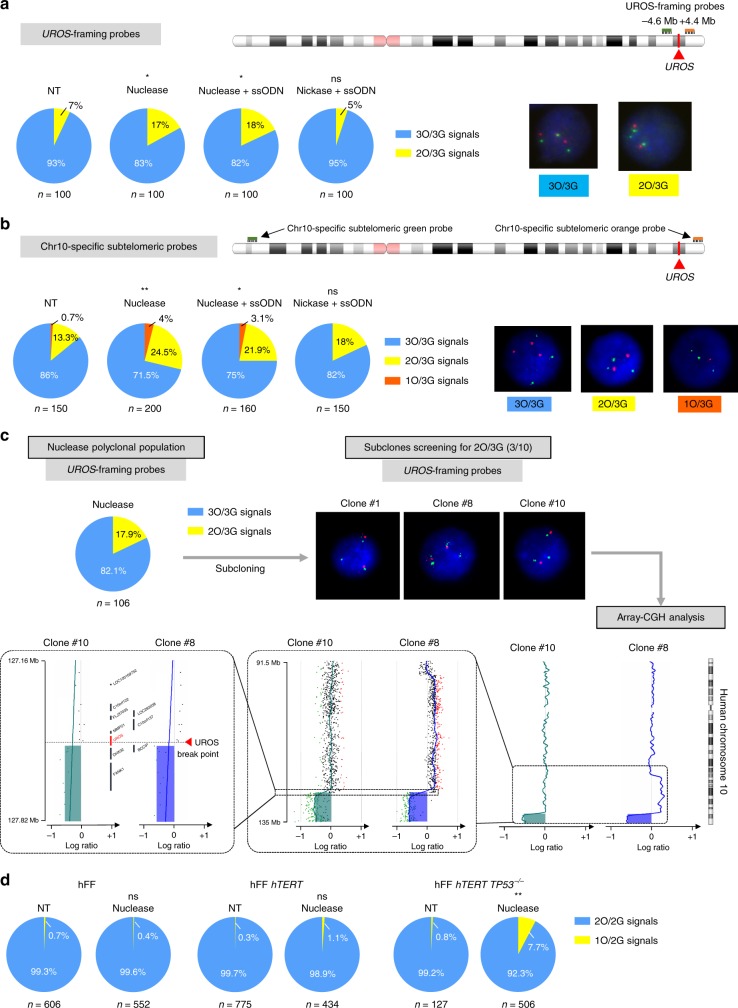
Unique DSB impairs chromosomal integrity. **a**, **b** DNA-FISH assay using (**a**) *UROS*-framing probes (respectively −4.6 Mb upstream and + 4.4 Mb downstream from *UROS* locus) or (**b**) Chr10-specific sub-telomeric probes for NT HEK293T cells or cells transfected with nuclease only, nuclease + ssODN or nickase + ssODN. For the two couples of probes, green (G) and orange (O) fluorescent probes are respectively upstream and downstream of the *UROS* gene. In this way, Chr10q terminal truncation is denoted by loss of orange signals. (Left) quantification of cells with 3 O/3 G, 2 O/3 G or 1 O/3 G signals. (Right) Illustrative DNA-FISH results for HEK293T. Statistical significance is inferred using two-sided chi-square test (versus NT cells). ns, not significant; *p < 0.05 **p < 0.01. **c** Array-CGH on truncated clones. First, HEK293T were transfected with nuclease and analyzed by FISH using *UROS*-framing probes. Transfected cells were subcloned and 3 out of 10 clones were identified as 2 O/3 G. Chromosome 10 integrity of clones #8 and #10 was evaluated by array-CGH. Deletion in #10: arr[GRCh37] 10q26.2q26.3(127516127_135404523)x2. Duplication and deletion in clone #8. arr[GRCh37] 10q24.1q26.2(95667790_127496056)x3~4,10q26.2q26.3(127516127_135404523)x2. **d** DNA-FISH assay using *UROS*-framing probes for primary wild-type fresh hFF (human Foreskin Fibroblasts) (hFF), immortalized with *hTERT* (hFF *hTERT*) or *TP53*^−^^/^^−^ immortalized fibroblasts (hFF *hTERT TP53*^−^^/^^−^), NT or transfected with nuclease. Quantification of 2 O/2 G and 1 O/2 G signals percentages. For (**a**, **b**, **c**), source data are provided as a Source data file

The HDR/indel ratio determined by next generation sequencing (NGS) is often considered as the gold standard to quantify the on-target genotoxicity of DSB. However, it uses PCR so it cannot reveal large chromosomal abnormalities. In view of recent publications describing that multiple DSBs induced by Cas9-nuclease can be used to eliminate an entire chromosome, we wondered whether chromosome 10 (Chr10) could be lost following a single DSB at *UROS* locus. To address this question, we performed fluorescent in situ hybridization (FISH) on HEK293T cells with a specific Chr10 centromere probe. We observed that HEK293T cells had mainly three Chr10 (69%), 25% had four Chr10 and 6% had only two Chr10 (Supplementary Figure 3b). Due to this chromosomal complexity, we analyzed blind a high number of interphase nuclei (at least 599 interphase nuclei per condition). Compared to non-transfected HEK cells, we did not observe any significant chromosomal loss of Chr10 in HEK293T cells transfected with nuclease co-delivered or not with ssODN (Supplementary Figure 3b). To evaluate the genome integrity around the *UROS* locus (locus 10q26.2), we performed another FISH analysis using probes framing *UROS* (a proximal probe at 4.6 Mb upstream *UROS*, labeled in green, and a distal probe 4.4 Mb downstream *UROS*, labeled in orange). Enlarged mapping of the location of probes is illustrated in Supplementary Figure 3a. Due to the chromosomal complexity of HEK293T cells observed, we decided to focus on cells apparently trisomic for Chr10, i.e., displaying three green signals (3 G profile) of the proximal probe. In these cells, we then counted the number of orange signals (distal probe) to evaluate 3 O/3 G or 2 O/3 G profiles, testing the absence or presence of distal deletion at *UROS* locus, respectively. In HEK293T cells transfected with nuclease, we observed a significant 10% increase in cells displaying a 2 O/3 G profile compared to non-transfected (NT) cells (17% of cells transfected with nuclease with a 2 O/3 G profile compared to 7% of NT cells with a 2 O/3 G profile, *p* < 0.05) (Fig. 3a). A similar result was observed in HEK293T cells transfected with nuclease and ssODN (18% of cells with a 2 O/3 G profile). These results could be consistent with a terminal deletion of Chr10 downstream of the *UROS* locus induced by nuclease-mediated DSB.

To confirm this hypothesis, we next hybridized the cells with one Chr10q sub-telomeric orange probe (Supplementary Figure 3a). We confirmed the complex pattern of Chr10 in HEK293T as revealed by a large analysis of a higher percentage of cells with only two Chr10q probes compared to non-transfected cells (9% in NT versus 22.6% and 20.1% in cells transfected with nuclease only or nuclease with ssODN, respectively, Supplementary Figure 4a). As previously, we then focused on HEK293T cells apparently trisomic for Chr10. To do so, we used another couple of probes: sub-telomeric probes at Chr10p-arm (labeled in green) and another one at Chr10q-arm (labeled in orange) (Fig. 3b) and focused on cells with a FISH profile with three green signals (3 G). The percentage of cells with a 3 O/3 G profile significantly decreased from 86% to 71.5 and 75% for nuclease without or with ssODN respectively in favor of cells with only two or one orange signals (from 14 to 28.5% and 25% for nuclease without or with ssODN respectively, *p* < 0.01). The use of sub-telomeric probes therefore allowed us to confirm that Chr10q truncation occurred in about 10% of HEK293T cells transfected with nuclease, without or with ssODN, resulting in a 7.5 Mb loss downstream of *UROS* (Fig. 3b). To check whether megabase-scale deletions could also occur upstream of *UROS* DSB, we compared the 3 O/2 G signal frequency between HEK293T cells non-transfected or transfected with nuclease. We did not observe any difference in 3 O/2 G signal frequency in two independent experiments (3.7 vs. 3.4% in first experiment and 7.6 vs. 8.5% in the second one respectively for NT cells and transfected with nuclease), suggesting that large deletions are mostly unidirectional downstream of *UROS* DSB.

To confirm the loss of 10q arm extremity and map the break point, we picked up 10 single clones of HEK293T cells previously transfected with nuclease. Using *UROS*-framing probes, FISH analysis revealed that 3 out of 10 clones lost one distal probe. We then performed high resolution array-Comparative Genomic Hybridization (CGH) (60 kb resolution) on two positive clones. Results demonstrated chromosomal truncation in both clones starting exactly from *UROS* DSB until the last probe near 10q chromosome telomere (50 kb from telomere) (Fig. 3c). Moreover, we observed in clone #8, upstream of *UROS*, a 31 Mb duplication with 50% of mosaicism. These data highlight the high genomic instability at the DNA break point with megabase–scale deletion or gain.

Altogether, these results strongly suggest that the editing strategy using a single DSB in one chromosome may induce megabase-scale chromosomal damage. To explore whether a similar effect occurs in other cell types, we transfected the K-562 hematopoietic cell line with nuclease and analysed outcomes using *UROS*-framing probes. Similarly, we found that nuclease transfection induced a significant increase in cells with only one signal of the probe distal to *UROS* (+ 11%), suggesting that chromosomal terminal deletion induced by nuclease-mediated DSB is not cell type-dependent (Supplementary Figure 4b).

HEK293T and K562 cell lines have a very complex and unstable karyotype and are deficient in p53 activity. To explore whether chromosome truncation could occur in primary cells, we repeated same experiments in primary human foreskin fibroblasts (hFF). Deep analysis of FISH assay using *UROS*-framing probes fortunately revealed that the percentage of 1 O/2 G cells does not increase after nuclease in fresh and *hTERT* (human telomerase reverse transcriptase) immortalized fibroblasts (Fig. 3d). Because FISH limit of detection is about 1%, we cannot exclude rare terminal truncations after nuclease transfection. We decided to use *hTERT* immortalized fibroblasts invalidated or not for the *TP53* gene. In contrast to *hTERT* immortalization alone, *TP53* knockout dramatically increased the risk, up to 10-fold (Fig. 3d). We demonstrate the strong involvement of p53 in Cas9 nuclease -induced chromosomal instability and highlight CRISPR-Cas9 nuclease genotoxicity in primary cells with inactivated p53.

### Cas9^D10A^ Nickase use prevents on- and off-target indels

**Fig. 4:**
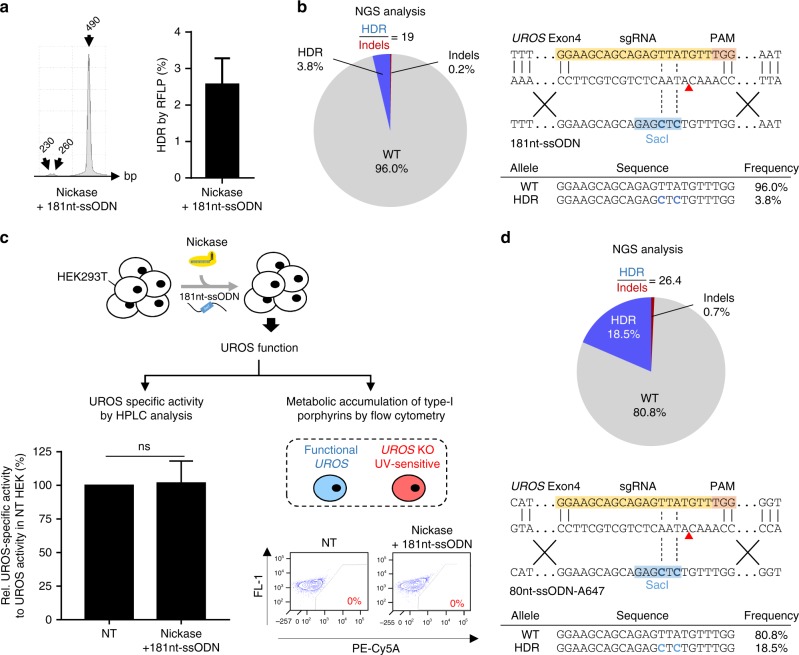
Single nickase-mediated HDR is associated with minimal indels leading to preserved UROS functionality. **a** (Left) Illustrative RFLP analysis and (Right) HDR frequency for HEK293T cells transfected with nickase and a 181nt-ssODN (*n* = 4). **b** (Left) NGS analysis of allelic outcomes and associated HDR/indel ratio following transfection of HEK293T cells with nickase and a 181nt-ssODN. (Right) Most common observed alleles (with frequencies ≥ 1%) aligned on the sgRNA sequence. HDR events in blue. **c** (Left) Quantification of UROS-specific activity from NT HEK293T cells or transfected with nickase and a 181nt-ssODN (*n* = 3). Values are normalized with NT cells. (Right) Illustrative flow cytometry results from NT cells or transfected with nickase and a 181nt-ssODN (*n* = 3). **d** (Top) NGS analysis of allelic outcomes and associated HDR/indel ratio following transfection of HEK293T cells with nickase and optimized amount of a 80nt-ssODN-A647. (Bottom) Most common observed alleles (with frequencies ≥ 1%) aligned on the sgRNA sequence. HDR events in blue. Results are presented as mean ± SEM. Data are from independent experiments. Statistical significance is inferred on raw data using two-tailed unpaired t-test for UROS-specific activity. ns, not significant. For (**a**, **c**), source data are provided as a Source data file

In view of the concerns about the Cas9-nuclease system, we wondered whether it was possible to abolish indels due to NHEJ and terminal chromosomal deletion by switching from DSBs to DNA single-strand breaks (SSBs). We switched to Cas9^D10A^-nickase (nickase) with the same gRNA and template. We obtained a modest but reproducible HDR rate as measured by restriction fragment length polymorphism (RFLP) (2.55% ± 0.73, *n* = 4) (Fig. 4a). PCR allelic analysis confirmed the HDR rate (2/38, 5.3%) and revealed the absence of alleles with on-target indels (0/38, Supplementary Figure 5 left). In parallel, we sorted 34 cellular clones and sequenced them. We observed that cells were either heterozygous (2/34) or not edited, but never with indels (Supplementary Figure 5 right). To increase sensitivity, we performed TIDER (Supplementary Figure 2b) and NGS analysis (Fig. 4b). Analyses found respectively 3.0% and 3.8% of HDR with only 0.2% of on-target indels with nickase compared to 64% with nuclease. This is therefore a very high HDR/indels ratio (19 vs 0.53 with nuclease). In contrast to nuclease, UROS enzymatic activity was maintained (101.8 ± 16%, *n* = 3, Fig. 4c left) without metabolic dysfunction (no fluorocytes, Fig. 4c right), demonstrating that the nickase approach does not impair gene function. Importantly, we did not detect any off-target indels in the predicted top 10 off-target sequences (Supplementary Table 1).

We hypothesized that the ssODN template concentration was a critical limiting factor for HDR efficiency. For this reason, we carried out ssODN dose-scaling and found that HDR frequency reached 5.1% at the highest concentration (2-fold higher with 5 µM vs 0.5 µM, *p* < 0.01, Supplementary Figure 6a). We then retained 5 µM as the optimal ssODN concentration for further experiments. To evaluate whether a shorter ssODN would facilitate ssODN transfection, we designed an 80nt-ssODN template. The HDR rate was similar but not improved (Supplementary Figure 6b). To monitor ssODN transfection efficacy, we used a ssODN coupled to an Alexa-647 fluorochrome (80nt-ssODN-A647). Surprisingly, we observed an unexpected increase in HDR rate without sorting A647-positive cells (+382%, compared to non-optimized condition, Supplementary Figure 6b).

We hypothesized that fluorochrome could stabilize the ssODN template and improve HDR. To confirm this hypothesis, we added 3 locked nucleic acid (LNA) bound in 5’ of the same 80nt-ssODN template and observed a similar increase in the HDR rate to the 5’ Alexa-647 chemical graft (+325%, compared to non-optimized condition, Supplementary Figure 6b). With all these improvements (high concentration of stabilized ssODN), TIDER analysis validated the high frequency of *SacI* (12.2% of alleles) and with a very low level of indels (1.2%) (Supplementary Figure 2c). NGS confirmed the 18% HDR rate and, most importantly, still with a minimal rate of indels (0.7%, Fig. 4d). Moreover, due to the minimal indels, other alleles were not modified (80.8% are WT vs 1.3% with nuclease).

To test whether single nickase could be used to edit other cell types, we transfected the K-562 cell line with the same optimized tools targeting *UROS* exon 4. We obtained a high rate of HDR (9.47 ± 2%, *n* = 6) measured by RFLP and confirmed by NGS analysis (8.5%, Supplementary Figure 7a, b). Again, a high HDR/indels ratio up to 14.1 with only 0.6% of indels and no UROS dysfunction (absence of fluorocytes, Supplementary Figure 7c) were observed. To confirm that this was not specific to exon 4, we targeted exon 10 of *UROS* near to the frequent c.683 C > T point mutation (p.Thr228Met)^18^ and transfected a 75nt-ssODN-A647 template together with the nickase (Supplementary Figure 8a). Similarly, we confirmed the editing by RFLP (Supplementary Figure 8b) without any UROS dysfunction (absence of fluorocytes detected by flow cytometry (Supplementary Figure 8c)). These data demonstrate that the nickase strategy can be applied to other cell types and loci. Moreover, unlike nuclease, nickase allows editing and leaves the other alleles non-edited. The absence of indels made it possible to perform iterative editing to reach higher HDR rates. With successive transfections, we dramatically improved the HDR rate at two loci and in different cell types (Supplementary Figure 9).

### Cas9^D10A^ nickase use prevents chromosomal truncation

To explore whether SSB can also avoid karyotype abnormalities, we first performed DNA-FISH using a centromeric probe. As expected, no chromosomal loss was observed (Supplementary Figure 3b). Then we used the two *UROS*-framing probes. To be highlighted, after nickase and ssODN template transfection, the percentage of cells with 2 O/3 G signal was significantly lower than with nuclease ssODN (5 and 17% respectively, Fig. 3a, *p* < 0.01) and similar to non-transfected cells. Sub-telomeric 10q FISH analysis of transfected cells with nickase showed significantly fewer cells with 1 O/3 G or 2 O/3 G (18% vs 25% Fig. 3b) than with nuclease. Importantly, FISH revealed the same pattern as non-transfected cells. Therefore, unlike nuclease, single nickase leads to HDR with only rare indels and no detectable chromosomal truncations using FISH.

### CEP modeling with single nickase approach

**Fig. 5:**
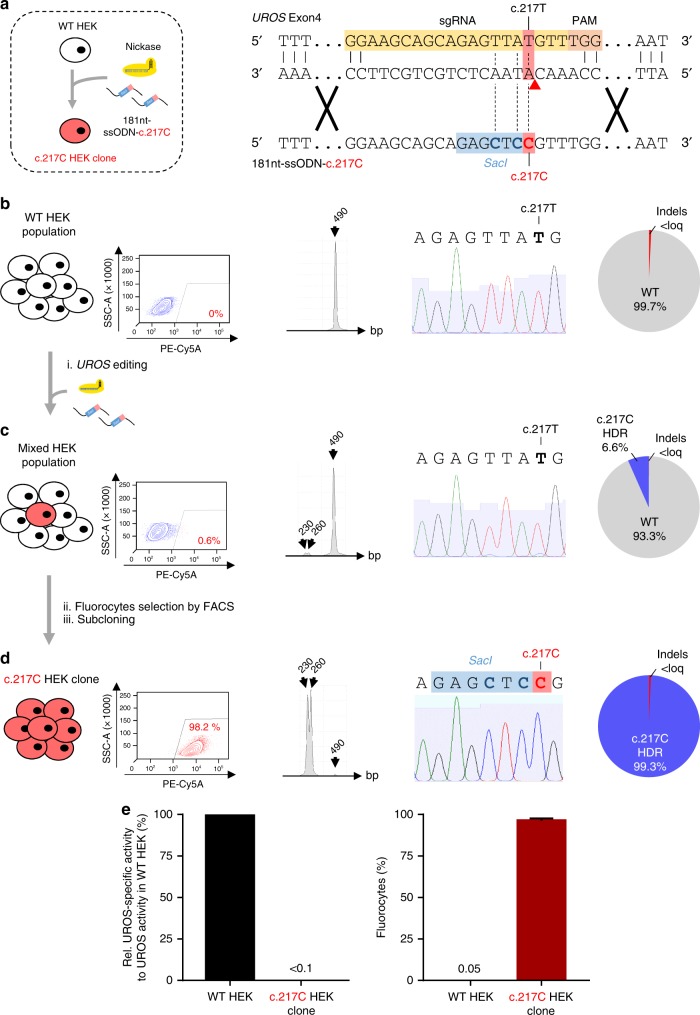
Single nickase-mediated gene editing results in c.217 C clone for CEP disease modeling **a** (Left) Scheme of gene editing approach to convert wild-type HEK293T (WT HEK) into homozygous c.217 C HEK clone using nickase and a 181nt-ssODN carrying c.217 C mutation (called 181nt-ssODN-c.217 C). (Right) Detailed view of exon 4 region and CRISPR-mediated HDR design using a c.217T-targeting sgRNA and a 181nt-ssODN-c.217 C carrying c.217 C mutation (red) in addition to silent *SacI* restriction site (blue). Expected cleavage position using nickase is indicated with a red arrow. **b**–**d** (From left to right) Illustrative flow cytometry results for fluorocyte analysis, representative RFLP analysis, sequence spanning *UROS* exon 4 c.217 position obtained by Sanger sequencing and indels and HDR quantification by TIDER analysis (**b**) for WT HEK, (**c**) for cells transfected with nickase and a 181nt-ssODN-c.217 C (Mixed HEK population) and (**d**) for sorted and subcloned fluorocytes (PE-Cy5A-positive), called c.217 C HEK clone. Loq: limit of quantification. **e** Characterization of c.217 C HEK clone. UROS functionality assay with (Left) quantification of UROS-specific activity and (Right) fluorocyte frequencies from WT HEK or c.217 C HEK clone. Values for UROS-specific activity are normalized against WT HEK. Results are presented as mean ± SEM. For (**e**), source data are provided as a Source data file

CRISPR-Cas9 is considered to be a powerful tool to model recessive genetic diseases. However, because of a high rate of indels concurrent to HDR, the probability of obtaining a mutant HEK293T homozygous for *UROS* c.217 T > C with a DSB approach is very low. In the light of our data, we tried a single nickase approach with a template containing the c.217 C mutation (181nt-ssODN-c.217 C) (Fig. 5a). RFLP, Sanger sequencing and TIDER analysis revealed that the editing event had occurred, leading to 6.6% of c.217 C HDR, without detectable indels and the appearance of 0.6% of fluorescent cells detected by flow cytometry (Fig. 5b compared to Fig. 5c). To investigate whether these fluorocytes resulted from the editing of the c.217 C mutation or from indels, we sorted the fluorescent cells. Thirty clonal allelic analyses confirmed that almost all the alleles (29/30) were edited with the c.217 T > C mutation and, importantly, without indels (Supplementary Figure 10). We then subcloned the sorted fluorescent cells and confirmed full editing in one clone with the T > C modification and *SacI* insertion (Fig. 5d). We obtained a fluorescent cell line homozygous for the c.217 C mutation (c.217 C HEK clone) with a strong decrease in UROS enzymatic activity (below 1% of the normal level) and therefore with fluorocytes due to the metabolic defect (Fig. 5e).

### CEP gene correction

**Fig. 6:**
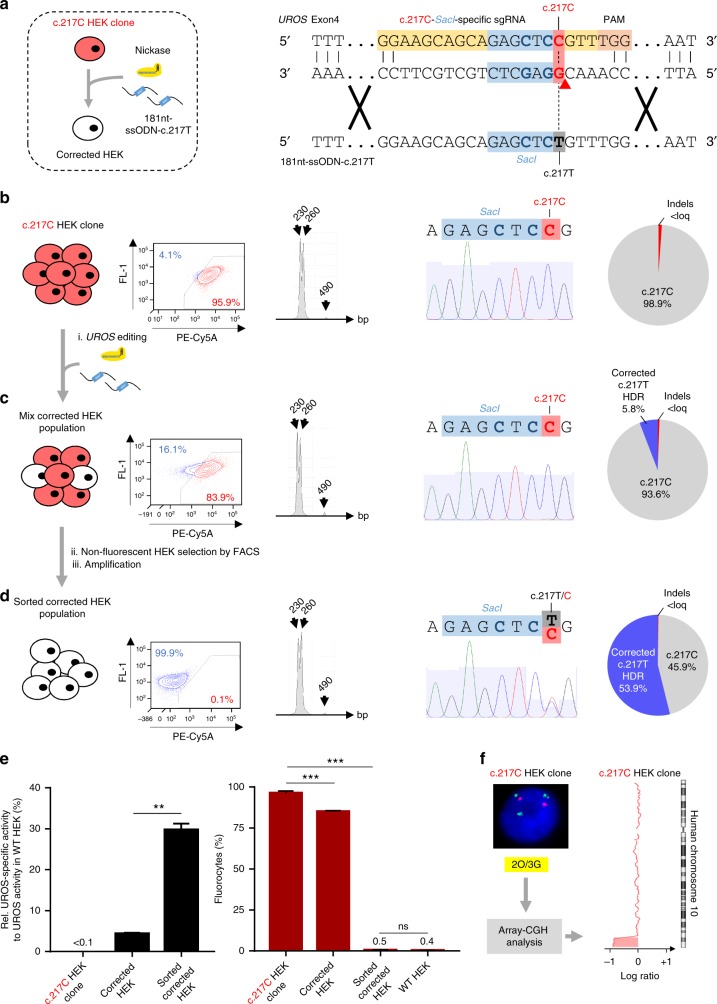
Single nickase-mediated gene editing allows precise genetic and phenotypic correction. **a** (Left) Scheme of gene editing approach to modify the c.217 C HEK clone and turn it into genetically and phenotypically corrected HEK using nickase and a 181nt-ssODN carrying the c.217 T correcting mutation (called 181nt-ssODN-c.217 T). (Right) Detailed view of the c.217 C HEK clone containing c.217 C mutation (red) and *SacI* restriction site (blue). Nickase-mediated HDR design using a c.217C-*SacI*-specific sgRNA and a 181nt-ssODN-c.217 T carrying the c.217 T correcting mutation (grey) in addition to silent *SacI* restriction site (blue). Expected cleavage position using nickase is indicated with a red arrow. **b**–**d** (From left to right) Illustrative FACS (fluorescent activating cell sorting)results for fluorocyte analysis (PE-Cy5A-positive), representative RFLP analysis, sequence spanning *UROS* exon 4 c.217 position obtained by Sanger sequencing and indels and HDR quantification by TIDER analysis, (**b**) for the c.217 C HEK clone, (**c**) for cells transfected with nickase and 181nt-ssODN-c.217 T (Mix corrected HEK population), and (**d**) for PE-Cy5A-negative HEK293T cells sorted by FACS (called Sorted corrected HEK population). Loq: limit of quantification. **e** UROS functionality assays with (Left) quantification of UROS-specific activity (*n* = 3) and (Right) fluorocytes frequencies from the c.217 C HEK clone, corrected HEK population and sorted corrected HEK population (*n* ≥ 3). Values for UROS-specific activity are normalized with WT HEK. Results are presented as mean ± SEM. Data are from independent experiments. Statistical significance is inferred on raw data using two-tailed unpaired t-test for UROS-specific activity and paired one-way ANOVA for fluorocyte frequencies; **p < 0.01 ***p < 0.001. **f** Chromosome 10 integrity of c.217 C HEK clone was analyzed by DNA-FISH using *UROS-*framing probes and by array-CGH. arr[GRCh37] 10q26.2q26.3(127458901_135404523)x2. For (**e**), source data are provided as a Source data file. ns, not significant

To test the ability of the single nickase approach to obtain genetic correction, we designed a novel sgRNA to specifically target the c.217 C mutant and *SacI* (c.217C-*SacI*-specific-sgRNA) (Fig. 6a). Next, we transfected the c.217 C HEK clone (Fig. 6b) with nickase and a 181nt-ssODN-c.217 T carrying the c.217 T correcting T-nucleotide. We obtained 5.8% of c.217 T HDR without indels, resulting in a slight decrease in fluorocyte percentage (Fig. 6c, e) that was correlated with a small rescue of UROS enzymatic activity (corrected HEK, 4.37% of WT UROS activity, in Fig. 6e). To enrich the corrected cell population, we sorted the non-fluorescent corrected cells (Fig. 6d). The presence of *SacI* as determined by RFLP and sequencing is proof that the non-fluorescent cells originated from cell sorting and not from contamination of the original WT cells (Fig. 6d). Sequencing showed c.217 C/T editing. About one-third of UROS activity was recovered in the sorted corrected cells (29.7% of WT UROS activity (Fig. 6e left panel), suggesting correction of 1 out of 3 alleles. This correction led to a full metabolic correction with the stable disappearance of porphyrins (at least up to one month) (Fig. 6e right panel). This is in agreement with published results showing that 10% of UROS activity is sufficient to avoid porphyrin accumulation *in vitro*^28^ and in vivo^29^. It is also in agreement with clinical observations of the absence of phenotypes or minor phenotypes in persons with genotypes inducing residual UROS enzymatic activity^30^. Using the single nickase approach, we obtained homozygous c.217 C CEP modeling (p.Cys73Arg) and a rescue of metabolic function. However, the intensity of Sanger sequencing peaks and TIDER demonstrated equal frequencies between c.217 T HDR and non-corrected c.217 C sequences and suggested that the corrected clone could be disomic and not trisomic for chromosome 10. To ensure that a terminal deletion had not occurred at the *UROS* locus, we performed FISH and array-CGH. Surprisingly, although there was no statistically significant difference in the 2 O/3 G FISH percentage between NT cells and cells transfected with nickase, we observed the loss of the 10q arm extremity starting at the *UROS* locus (Fig. 6f). This demonstrated that even with nickase, this event could rarely appear. We hypothesize that cell sorting based on the highest fluorescence could bias the results by enriching cells with chromosomal truncation (lower UROS activity after truncation than with mutation).

## Discussion

Additive gene therapies are successful in treating monogenic hematopoietic disorders^31–33^. However, transgenes semi-randomly integrate the hematopoietic stem cell (HSC) genome leading to a risk of genotoxicity. The aim of iPSC-based gene therapy is to control additive gene therapy by sorting cells with integration in a genomic safe harbor^29,34^. Editing a gene at its endogenous locus by removing or correcting deleterious mutations rather than adding a new transgene has the potential to improve disease modeling and to solve insertional mutagenesis and non-physiological gene regulation problems^3,35^. A high-fidelity HDR is a prerequisite for the safe use of CRISPR-Cas9-mediated gene therapy in the future and for obtaining reliably edited cell models of genetic diseases. Off-target modifications by the CRISPR-Cas9 first-generation nuclease have been widely reported^4,5,36–39^. In our work, we confirm lack of specificity of the nuclease.

Unlike the off-target one, the on-target NHEJ activated in response to DNA DSBs has often been underestimated. Using the CEP model, we show that this is a major limiting factor for PGE. Indeed, flow cytometry analysis revealed an unacceptably high percentage of fluorocytes due to a profound deficiency of UROS enzymatic activity. NGS analysis demonstrated the predominant indels on-target caused by the NHEJ following nuclease-mediated gene editing. To improve the HDR/indel ratio, it is possible to insert a positive selection cassette in the donor template. However, that may cause genotoxicity by transgene integration and an immune response against the exogenous selection protein. Other techniques have been proposed: i) induce HDR activation using RAD51 agonist^40^ or cell synchronization to promote HDR during S phase;^41^ ii) inhibit the NHEJ by DNA ligase IV^42,43^, KU70^44^, 53BP1 inhibitors^45^ or downregulation of polymerase θ^46,47^. Nevertheless indels remain when using these pharmacological approaches, and inhibition of the genotoxicity DNA repair pathways has to be evaluated on primary cells. Indeed, using a zinc-finger-mediated DSB approach to target *MLL*, Do et al.^48^. reported that inhibition of the NHEJ by the DNA-PK inhibitor increases chromosomal translocations.

Our study reveals a safety concern regarding the medical perspectives of CRISPR-Cas9 editing. Indeed, using two different cell lines and immortalized *TP53* KO fibroblasts, we observed a high frequency of chromosomal terminal deletions after only one nuclease-mediated DSB (10% in cancer cell lines and 7.7% in fibroblasts). This truncation is not due to a second cut that destabilizes 10q arm because none of the top-10 off-targets are in chromosome 10. This megabase-scale terminal deletion (7.5 Mb) is larger than the kilobase-scale deletions recently described by Adikusuma et al. in mouse zygotes^15^. Array-CGH is required to observe these deletions because of the loss of primer sequences prevents PCR amplification. It confirms the full truncation of the 10q arm extremity. This damage caused by DSB could lead to severe outcomes in clinical applications. This truncation is critical not only for UROS activity but also for the elimination of multiple downstream genes and telomeres. Given the hypothesis that tumor-suppressor genes or genes involved in DNA repair are lost with the chromosome fragment, this could confer a selective advantage to damaged cells. In our example, 10q terminal deletion eliminated 43 genes (Supplementary Figure 11). Among them, five are proto-oncogenes and seven are tumor suppressors. Alarmingly, five of the tumor suppressors have been reported in leukemia (*BCCIP*^49^*, PTPRE*^50^*, MGMT*^51^*, BNIP3*^52^*, EBF3*^53^*)*. Therefore, before any clinical applications are envisaged, it will be mandatory to evaluate this oncogenic risk in primary hematopoietic cells to avoid grafting of leukemia-prone cells, as previously observed with additive gene therapy clinical trials using the first generation of oncoretroviral vector^54^. Surprisingly, array-CGH revealed in one clone a mosaic duplication upstream of *UROS* DSB, which testifies to the complexity of DSB repair pathways. This unexpected rearrangement linking duplication with terminal deletion has already been described^55^.

Fortunately, we did not observe any entire loss of chromosome 10. However, this risk has to be considered when two or more cuts by nuclease are needed. Indeed, a recent paper reported that only two cuts at the centromere (but not in the long arm) of chromosome Y induced its loss in mESC^18^. Two recent papers in the field of gene therapy recently proposed to excise alpha-globin enhancer^56^ or a long fragment of 13.6kB with a putative gamma-delta intergenic fetal hemoglobin silencer using two cuts framing the locus^57^. In both papers, they obtained deletion or inversion of the targeted region. In light of these data and others^17,18^, a careful evaluation of chromosomal integrity will be required before these approaches can be considered as entirely safe.

Two recent papers highlight the adverse role of p53 for Cas9-mediated editing^58,59^. They showed that CRISPR-Cas9 genome editing induced a transient p53-mediated DNA damage response and G1 cell-cycle arrest in immortalized retinal pigment endothelium cells and human pluripotent stem cells, respectively. Inhibition of p53 increases HDR efficiency and cell viability. Edited live cells are selected by their low-functional p53 pathway. This suggest that inhibition of p53 in edited cells could increase cell vulnerability to tumorigenic mutations or chromosomal rearrangements. In our study, we observed a serious side-effect of Cas9 nuclease, i.e. terminal chromosomal deletion in HEK293T and K-562 cell lines. These immortalized cells are known to have their p53 function inhibited^60,61^. This probably explains the high HDR rate obtained in our study and their vulnerability to chromosomal extremity losses. Chromosomal rearrangements in primary cells could be promoted depending on their p53 status. We demonstrate the strong involvement of p53 in chromosomal instability-induced CRISPR-Cas9 with a dramatic increase in truncation rate in *TP53* KO primary cells. Taken together, these data highlight the vulnerability of primary cells with p53 dysregulations. It will be critical to evaluate and monitor this risk in hematopoietic stem cells and embryos before pre-clinical applications of CRISPR-Cas9 nuclease.

In our laboratory, we have already used mouse models to demonstrate the efficacy of additive gene therapy in vitro and in vivo for CEP^29,62,63^. The clinical severity of the disease and the lack of specific treatment, apart from bone marrow (BM) transplantation with an HLA-compatible donor, are strong arguments for gene therapy. DNA sequence damage induced by the NHEJ repair system and karyotype abnormalities using nuclease underline the need to use the CRISPR-Cas9 approach for precise genome editing with caution.

Nicks (SSBs) were long thought to undergo immediate ligation that prevented them from initiating editing. Many believed that nicks could only initiate recombination if they were converted to DSBs. We now have evidence of physiological nick-initiated HDR. For example, once the initial nick is generated, RAG1/2 (Recombination-activating gene) proteins are implicated in VDJ recombination in the adaptive immune system^64^. The proof-of-principle that HDR can be obtained following DNA SSB was first obtained with zinc finger nuclease technology using a DNA donor template with 1–4% efficiency. However, it was 6-fold less efficient than with DSB^65^. The structure of the exogenous repair donor seems to determine the HDR pathway. ssODN have attracted considerable interest as they are cheap to synthesize, easy to use, short-lived and cannot enter the genome. Recent publications^22,66^, demonstrate that HDR by ssODN may proceed via an alternative repair pathway. It is currently thought that this process depends on whether ssODN is i) complementary to the nick and serves as a matrix to synthesize the edited strand, or ii) complementary to the intact strand of the target and directly fills the nick gap. In both cases, HDR efficiency seems to increase when RAD51 or BRCA2 are inhibited. Interestingly, the base excision repair pathway (BER) but not NHEJ repairs single nicks with high fidelity^67^. The proof of concept with nickase was made more recently^22^ but still with low efficiency, thus limiting its interest for clinical applications. We demonstrate that a limiting factor for efficient HDR with SSB is the ssODN concentration and stabilization. Moreover, minimal indels allow iterative transfections to reach the same HDR efficiency as nuclease. This promising method that produces high PGE with minimal indels will provide an important boost to CRISPR-Cas9-mediated gene therapy.

Using CEP disease as a proof of concept, we demonstrate that the single nickase method is the best for homozygous mutation modeling. It allows PGE at the locus with all regulatory elements of the promoters and an ideal physiological enzymatic rescue. Importantly, this approach is devoid of off-target indels and should be preferred to nuclease approaches. Moreover, it strongly reduced genotoxic terminal chromosomal deletion mediated by DSB in two cell lines. The next step will be to evaluate it for PGE in hematopoietic stem/progenitor cells in terms of feasibility and to measure the chromosomal abnormality rate as compared to nuclease. It could be helpful for the development of future clinical trials in hereditary hematopoietic disorders. The current findings pave the way for corrective gene therapy with single nickase, demonstrating it to be a safer alternative genome editing technology with high efficiency and better fidelity. However, truncation may rarely occur even with single nickase, as shown by our monocellular analysis. The low frequency of these rearrangements meant that it was very difficult to detect them in a polyclonal population. Alarmingly, both DSB and SSB may induce complex chromosomal rearrangements. Genome editing without any DNA break such as using CRISPR base editors could be a promising alternative.

## Methods

### Cell culture and transfection

Human embryonic kidney (HEK) cell line HEK293T (ATCC®, Manassas, VA, USA) was maintained in Dulbecco’s modified Eagle’s medium (DMEM), low glucose (1 g.L-1), L-Glutamine (1 g.L-1) and pyruvate (Gibco® by Lifetechnologies^TM^, Carlsabad, CA, USA) supplemented with 10% fetal bovine serum, 100 U/mL penicillin, and 100μg/mL streptomycin (all from Eurobio, Courtaboeuf, France). K-562 cell line (ATCC®) was maintained in RPMI Medium 1640, l-Glutamine, 25 mM HEPES (Gibco®) supplemented with 20% fetal bovine serum, GlutaMAX (Gibco®), 100 U/mL penicillin, and 100 μg/mL streptomycin. Human foreskin fibroblasts (HFF) were from a healthy person in accordance with the ethical standards of the committee responsible for human experimentation (Centre Hospitalier Universitaire de Bordeaux). Skin fragments were treated with trypsin-EDTA for 3 h at 37 °C and fibroblasts were isolated. *hTERT* immortalized fibroblasts were from ATCC® (CRL 4001, BJ-5ta), invalidated or not for *TP53* by CRISPR-Cas9. Fibroblasts were maintained in the same culture medium as HEK293T. Both cell lines were cultured at 37 °C, 5% CO_2_ in a humidified chamber.

### Editing tools

Cells were transfected by electroporation using the Nucleofector AMAXA electroporation system (Lonza®, Bale, Switzerland). In brief, 10^6^ HEK293T (or K-562) cells were nucleofected with 200 ng (or 4 µg) of nuclease- or nickase-containing plasmid and 0.05 µM of specified ssODN. ssODN optimized concentration was used when specified and corresponded to 1.7 µM. Cells were then seeded onto 6-well plates (Corning©, Tewksbury, MA, USA) and cultured as described above. Transfected cells were then positively selected 24 h after transfection either by addition of 1.25 μg mL^−1^ puromycin (Sigma-Aldrich, Saint Louis MO,USA) for 36 h or by GFP-positive selection by Fluorescent activating cell sorting (FACS) using BD FACS Aria®.

For fibroblasts, 4D-Nucleofector system was used with P3 Primary Cell Line 4D-Nucleofector® in association with CZ-167 program. In brief, 200,000 hFF were nucleofected with 16.9 µg Cas9 RNP and 5 µM of Alt-R® Cas9 Electroporation Enhancer. To form RNP, Cas9 protein was complexed to crRNA:tracrRNA according to the manufacturer’s instructions. Then complexes were incubated for 20 min at room temperature before electroporation. Cas9 protein (Alt-R® S.p. Cas9 Nuclease V3), crRNA (Alt-R® CRISPR-Cas9 crRNA) were purchased from Integrated DNA Technologies.

Nuclease-containing plasmid was a modified version of pSpCas9n-2A-PuroR (pX462) obtained from Addgene (Cambridge, MA, USA) (#48141). First, to switch from Cas9D10A to Cas9, an insert containing the CBh promotor and 253 bp of Cas9 was obtained by *XbaI* and *BgIII* digestion of pX330 (Addgene, #42230), and then ligated in pX462 digested with the same enzymes. Then, the puromycin-resistance gene (*pac*) was replaced by a human codon-optimized version (PuroR-HO). sgRNA was cloned into pUC19 obtained from Addgene (#50005) using the *BbsI* restriction site and co-delivered at transfection with the nuclease-containing plasmid.

The nickase-containing plasmid was also a modified version of pX462 with a human codon-optimized version of the puromycin-resistance gene (PuroR-HO). The nickase-containing plasmid used for GFP-positive selection was pSpCas9n(BB)−2A-GFP (PX461), Addgene (#48140). In both nickase-containing plasmids, sgRNA was directly added using the *BbsI* restriction site according to the Feng Zhang protocol (Addgene). All sgRNAs were designed using the CHOPCHOP v2 algorithm^68^ (chopchop.cbu.uib.no) and based on a unique sequence with 20 nucleotides. All ssODN templates used in the study were purchased from Integrated DNA Technologies (IDT, Coralville, IA, USA). For 80nt-ssODN-A647 and 75nt-ssODN-A647, an Alexa Fluor® 647 was chemically linked at the 5’ terminal end to ssODN by NHS Ester link. For 80nt-ssODN-LNA, three Locked Nucleic Acids were added at the 5’ terminal end.

### Flow cytometry for fluorocyte quantification and sorting

Fluorocyte quantification and sorting for disease modeling and correction was performed by flow cytometry. UV-sensitive type-I porphyrins were excited at 496 nm and the emitted wavelength was approximately 667 nm, detected by the PE-Cy5A PMT channel (FACSCanto, BD, Franklin Lakes, NJ, USA). FL-1 is a control green fluorescent channel used to exclude autofluorescent cells. Cells were sorted by BD FACS Aria®.

### RFLP for HDR quantification

Genomic DNA was extracted using Nucleospin® Tissue (Macherey-Nagel, Duren, Germany) according to the manufacturer’s protocol. The genomic region flanking *UROS* exon 4 (or exon 10) was amplified by PCR (HotStarTaq Plus DNA polymerase, Qiagen®, Venlo, Netherlands) with adequate primers (Supplementary Table 2). PCR products were purified with Nucleospin® Gel and PCR Clean-up (Macherey-Nagel) and digested with *SacI* (or *ApaI* for exon 10 *UROS* analysis) restriction enzyme (New England Biolabs, Ipswich, MA, USA) for 1 h at 37 °C. Then, 5 ng digestion products were loaded into the Agilent® 2200 TapeStation (Santa Clara, CA, USA) capillary electrophoresis using D1000 ScreenTape and D1000 reagents according to the manufacturer’s protocol. Quality control of enzymatic digestion efficiency is included in each assay.

### Allelic and cellular clonal analysis

For allelic analysis, purified PCR products were sub-cloned into a “TOPO TA Cloning” vector (Invitrogen, Life Technologies® (Carlsbad, CA, USA) according to the manufacturer’s protocol. Electro-competent bacteria (Stbl3™) (Thermo Fisher Scientific, Waltham, Massachusetts, USA) were transformed with the TOPO and colonies were analyzed by Sanger sequencing after PCR. For cellular clonal analysis, transfected cells were isolated by the limiting dilution method and each clone was analyzed by Sanger sequencing after PCR. Sanger sequencing was done on purified PCR products and sequenced by LIGHTRUN (GATC Biotech, Konstanz, Germany).

### UROS enzymatic activity and metabolic correction

UROS activity was determined by an enzyme-coupled assay as described previously^69^. Briefly UROS activity was determined by an enzyme-coupled assay. For that, porphobilinogen was first converted to hydroxymethylbilane, the natural substrate for UROS, by hydroxymethylbilane synthase. Then, the uroporphyrinogen reaction products were oxidized to their respective uroporphyrin isomers, which were then resolved and quantitated by reversed-phase high-pressure liquid chromatography. One unit was defined as the amount of enzyme that formed 1 nmol of uroporphyrinogen III per hour at 37 °C.

### TIDER analysis and NGS-Deep sequencing for allelic outcomes

Tracking of Insertions, DEletions and Recombination events (TIDER)^28^ was used to determine HDR and indels frequencies. 490pb PCR product (carrying c.217 T) from non-transfected HEK293T was provided as control chromatogram. For Fig. 6, 490pb PCR product carrying the c.217 C mutation was used as control. Reference chromatogram with either *SacI*-c.217 C (Fig. 5) or *SacI*-c.217 T (Fig. 6 and Supplementary Figure 2) were obtained from allelic analysis (Supplementary Figure 5 and Supplementary Figure 10). Defaults parameters were conserved for analysis. The limit of quantification (loq) was defined at 2%.

Genomic DNA was extracted using Nucleospin® Tissue (Macherey-Nagel®) according to the manufacturer’s protocol. The genomic region flanking *UROS* exon 4 was amplified by PCR (KAPA HiFi DNA polymerase, Kapa Biosystems®, Cape Town, South Africa) with adequate primers (Supplementary Table 2). PCR products were purified with Nucleospin® Gel and PCR Clean-up (Macherey-Nagel®). To prepare sequencing libraries, the Illumina Nextera XT Kit (Illumina®, San Diego, California, USA) was used and nested-PCR using Illumina primers was performed on purified PCR products. An Illumina MiSeq instrument (Illumina®) was used for high-throughput sequencing. The average depth of each genome analysis was 10,000. Quality of paired-end reads was checked with FastQC (Galaxy, https://usegalaxy.org/). Then, data were analyzed using the CRISPR Data Analysis and Visualization (CRISPR-DAV) pipeline^70^.

### Cytogenetic examination of chromosome 10

FISH was performed on interphase nuclei of HEK cells or K-562 cell line, with probes targeting the following regions on chromosome 10: centromeric region (XCE 10 probe, labeled in orange) (MetaSystems Probes, Altlussheim, Germany), locus 10q26.11 (BAC RP11–79M19 probe, labeled in green) (Empire Genomics, Buffalo, NY, USA), locus 10q26.2 (BAC RP11–31M22 probe, labeled in orange) (Empire Genomics, Buffalo, NY, USA), sub-telomeric regions (p-arm and q-arm sub-telomere probes, respectively labeled in green and orange) (Cytocell Ltd, Cambridge, UK). Preparations were pre-treated as indicated below. Briefly, the slides were successively immersed in a 2xsaline-sodium citrate buffer for 10 min at 37 °C, in a 0.01% pepsin solution for 10 min at 37 °С, in a 1× phosphate-buffered saline (PBS) solution for 5 minutes, in a 3.7% formaldehyde solution for 5 min, and in a 1xPBS solution for 5 minutes. FISH probes and DNA were then co-denaturated according to the manufacturers’ protocols, and hybridization was performed overnight at 37 °C. The slides were then successively immersed in wash solutions and the nucleic acids were counterstained by 4,6-diamidino-2-phenylindole. The slides were then placed under an Axio Imager2 microscope with an epi-fluorescence source (Carl Zeiss AG, Oberkochen, Germany). The microscope was linked to the Metafer4 software for automated image acquisition and processing (MetaSystems GmbH, Altlussheim, Germany).

Genomic DNA was extracted with the Wizard Kit (Promega Corporation, Madison, USA) following the protocol validated in the laboratory.

### Off-target analysis

For sgRNA targeting exon 4 *UROS* locus, the Top 10-predicted off-target sites, identified by CRISPOR software were amplified in genome-edited HEK293T and subjected to Sanger sequencing, followed by comparison to non-transfected cells by TIDER analysis.

Primers used for off-target analysis are in Supplementary Table 2.

### Array-CGH

Array-CGH was performed on 8 × 60k oligonucleotide microarrays (Agilent Technologies, CA). DNA was labeled (cyanine 3 or cyanine 5) using the Genomic DNA ULS Labeling Kit from Agilent Technologies and hybridized onto the microarrays according to the manufacturer’s instructions (Agilent). Scanning of the microarrays was performed using a G2565CA scanner (Agilent). Data analysis was carried out with Agilent Technologies software, namely Feature Extraction for Cytogenomics V5.0 for the fluorescence ratio calculation and Agilent CytoGenomics 3.0.1.1 to visualize chromosomal imbalances. Deletions and duplications in the heterozygous state were characterized by values of the log2 ratio of fluorescence intensities (cyanine5/cyanine3) below −0.5 and above + 0.3, respectively, with the statistical algorithm ADM2 used at a threshold of 5.

### Statistics

Statistical significance was inferred when necessary. Graph Pad Prism 6 software was used for statistical analysis. Results are presented as mean ± SEM (standard error of the mean). The two-tailed unpaired *t* test was done to compare means of two groups. One-way ANOVA, completed with unprotected Fisher’s Least Significant Difference test, was used to compare three groups. Two-tailed Khi2 tests were used to compare percentages. All comparisons are shown with black bars. Null hypothesis was rejected when *p* value < 0.05. *p < 0.05; **p < 0.01; ***p < 0.001; ns, non-significant.

### Reporting summary

Further information on experimental design is available in the Nature Research Reporting Summary linked to this article.

## Supplementary information

Supplementary Information

Reporting Summary

Source Data

## Data Availability

All next-generation sequencing data sets have been deposited in the NCBI database under BioProject accession no. PRJNA521053 with associated BioSample no. SAMN10877893 for HEK293T NT and BioSample no. SAMN10877894, no. SAMN10877895 and no. SAMN10877896 for HEK293T respectively transfected with Nuclease + 181nt-ssODN (Fig. 2b), Nickase + 181nt-ssODN (Fig. 4b) and Nickase + 80nt-ssODN-A647 (Fig. 4d). Reads are available at Sequence Read Archive database (accession number no. SRR8534354, no. SRR8534355, no. SRR8534352 and no. SRR8534353 for HEK293T NT and HEK293T respectively transfected with Nuclease + 181nt-ssODN, Nickase + 181nt-ssODN and Nickase + 80nt-ssODN-A647). The data that support the findings of this study are available from the corresponding author upon reasonable request. Source data for bar charts and pie charts presented in Figs. 2, 3, 4, 6 and 6 as well as Supplementary Figures 3, 4, 6, 7, 8, 9 are provided with the paper.
