# Supplementary material for: CRISPR-Cas9 genome editing induces megabase-scale chromosomal truncations
Source: Nat Commun. 2019 Mar 8;10:1136. doi: 10.1038/s41467-019-09006-2 (PMC6408493; doi:10.1038/s41467-019-09006-2)
Supplement: Supplementary file 1 — Supplementary Information [file 41467_2019_9006_MOESM1_ESM.pdf]

CRISPR-Cas9 genome editing induces megabase-scale chromosomal truncations

Cullot et al.

Supplementary Figure 1

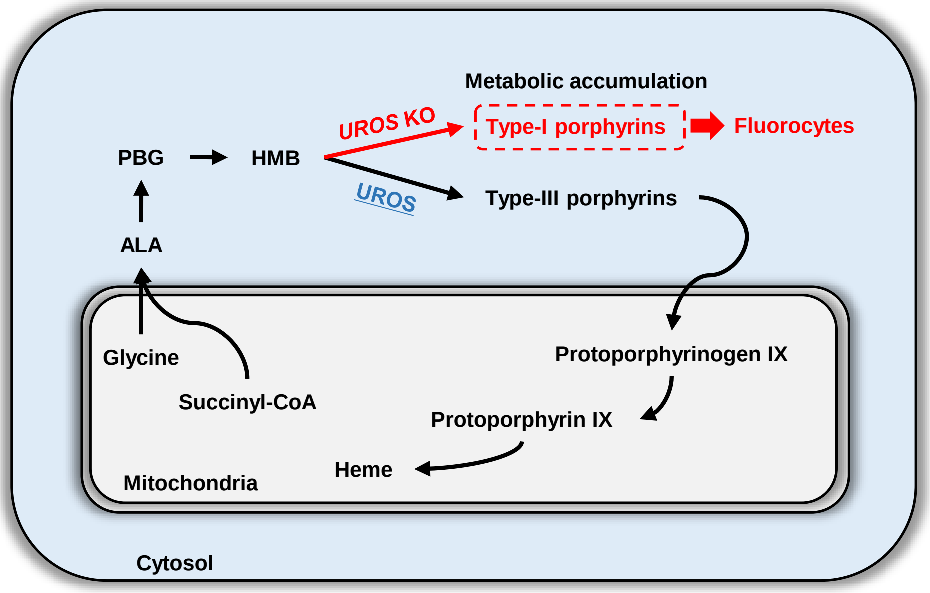

Supplementary Figure 1: Heme biosynthesis pathways is impaired in congenital erythropoietic porphyria. In physiological conditions, the uroporphyrinogen III synthase (UROS) catalytically cyclizes the hydroxymethylbilane (HMB) into type-III porphyrins leading to heme synthesis. In *UROS* KO cells as in congenital erythropoietic porphyria (CEP), HMB spontaneously cyclizes into type-I porphyrins which enter a metabolic impasse, accumulate and turn the cells into UV-sensitive cells called fluorocytes (PE-Cy5A-positive). CoA, Coenzyme A ; ALA, δ-aminolevulinic acid ; PBG, Porphobilinogen ; HMB, Hydroxymethylbilane.

Supplementary Figure 2

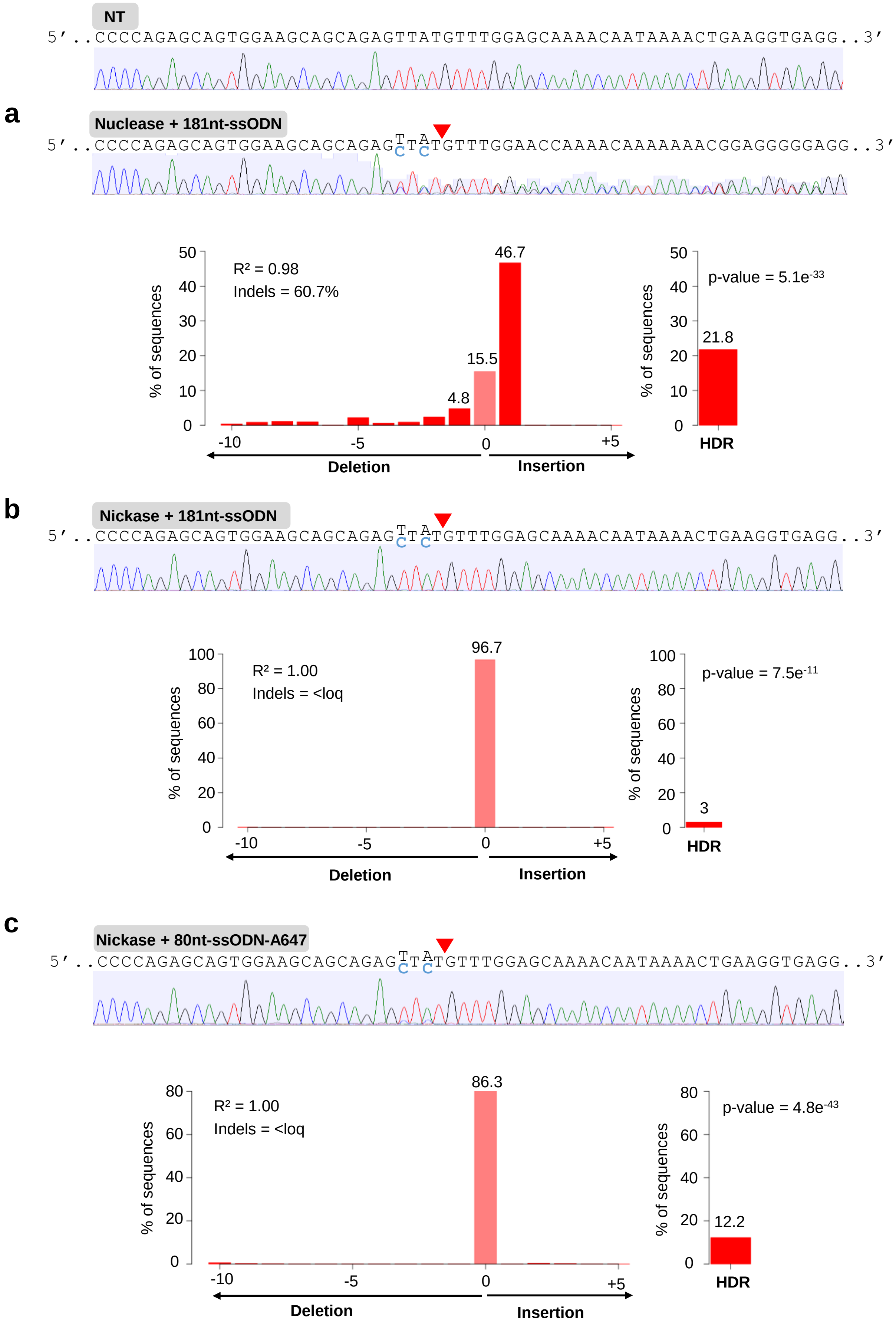

Supplementary Figure 2: TIDER analysis of gene editing. (Top) UROS exon 4 Sanger sequencing of non-transfected (NT) HEK293T used as the reference sequence for TIDER analysis. a,b,c, HDR and indel quantification for cells transfected a, with nuclease and a 181nt-ssODN template, b, with nickase and a 181nt-ssODN template and c, with nickase and optimized amount of a 80nt-ssODN-A647 template. Expected cleavage position is indicated with a red arrow. HDR events in blue.

Supplementary Figure 3

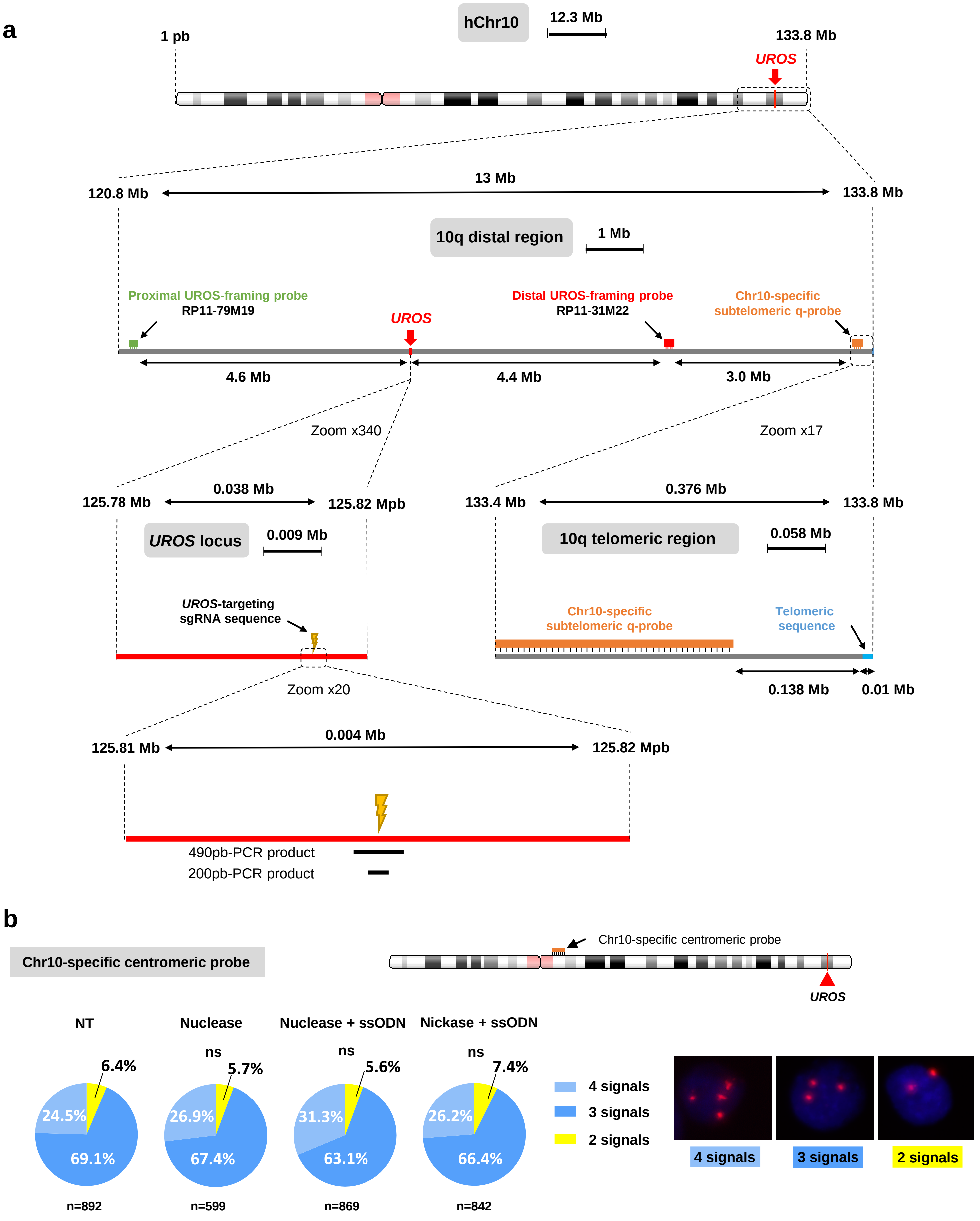

Supplementary Figure 3: a, Size and chromosomal location of FISH probes and PCR products on chromosome 10, with enlarged pictures of *UROS* locus and 10q telomeric region. **b**, DNA-FISH assay using Chr10-specific centromeric probe, for NT HEK293T or transfected either with nuclease, nuclease + ssODN or nickase + ssODN. (Left) Quantification of cells with 2, 3 or 4 orange signals. (Right) Illustrative FISH results for HEK293T with 2, 3 or 4 signals. For (b), source data are provided as a Source data file.

Supplementary Figure 4

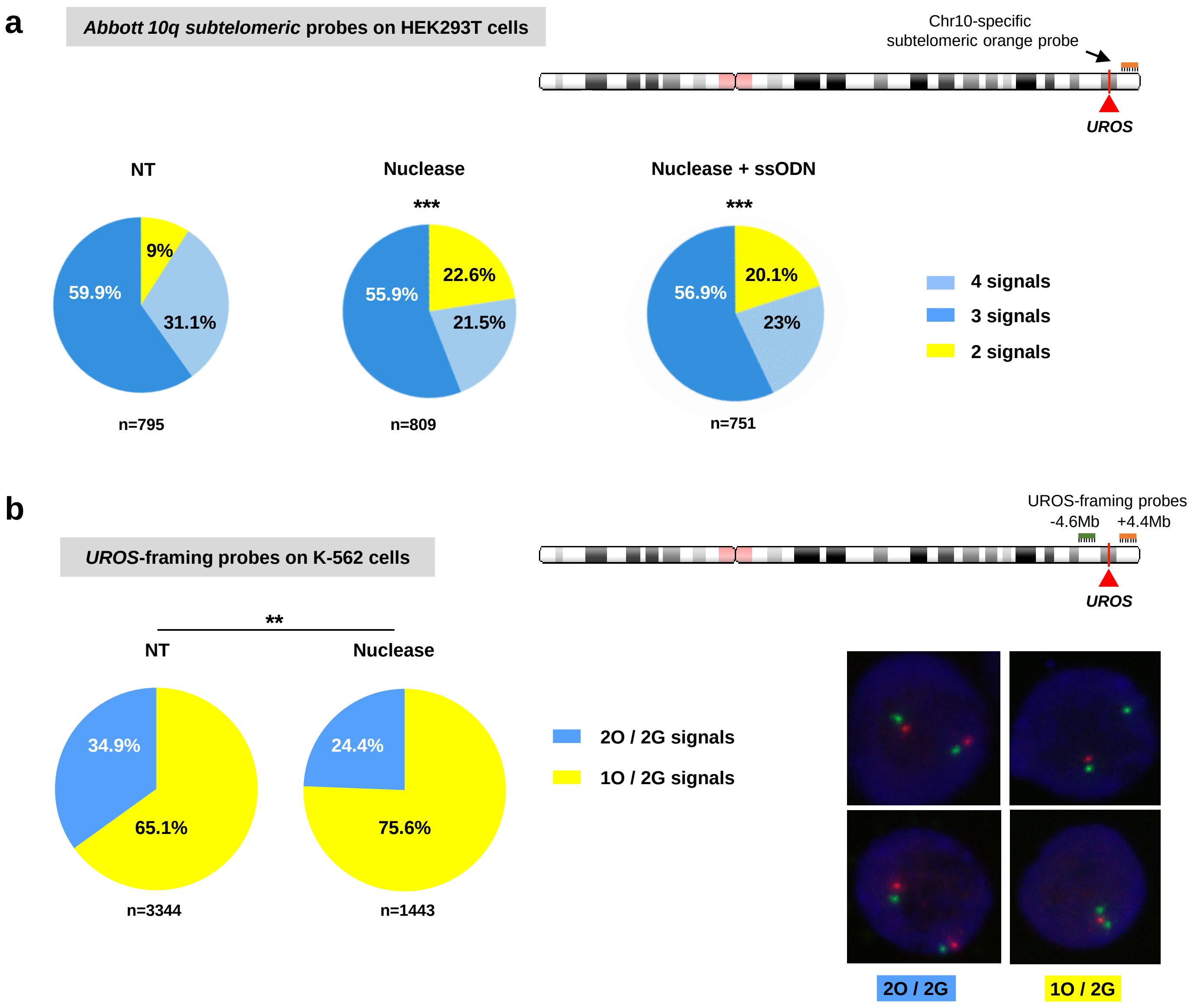

Supplementary Figure 4: Nuclease-mediated gene editing impairs chromosomal integrity in HEK293T and K-562 cells. a, Quantification of cells with 2, 3 or 4 orange signals by DNA-FISH assay using Abbott 10q sub-telomeric orange probe in HEK293T cells. **b,** Loss of orange signals is quantified by DNA-FISH assay using *UROS*-framing probes (shown in detailed view of *UROS* locus) for non-transfected K-562 or transfected with nuclease. (Left) Quantification of cells with 2O/2G or 1O/2G profiles. (Right) Illustrative FISH results for K-562 with 2O/2G or 1O/2G profiles. Green and orange fluorescent probes are respectively -4.6Mb upstream and +4.4Mb downstream *UROS* gene. **; p<0.01. For (a) and (b), source data are provided as a Source data file.

Supplementary Figure 5

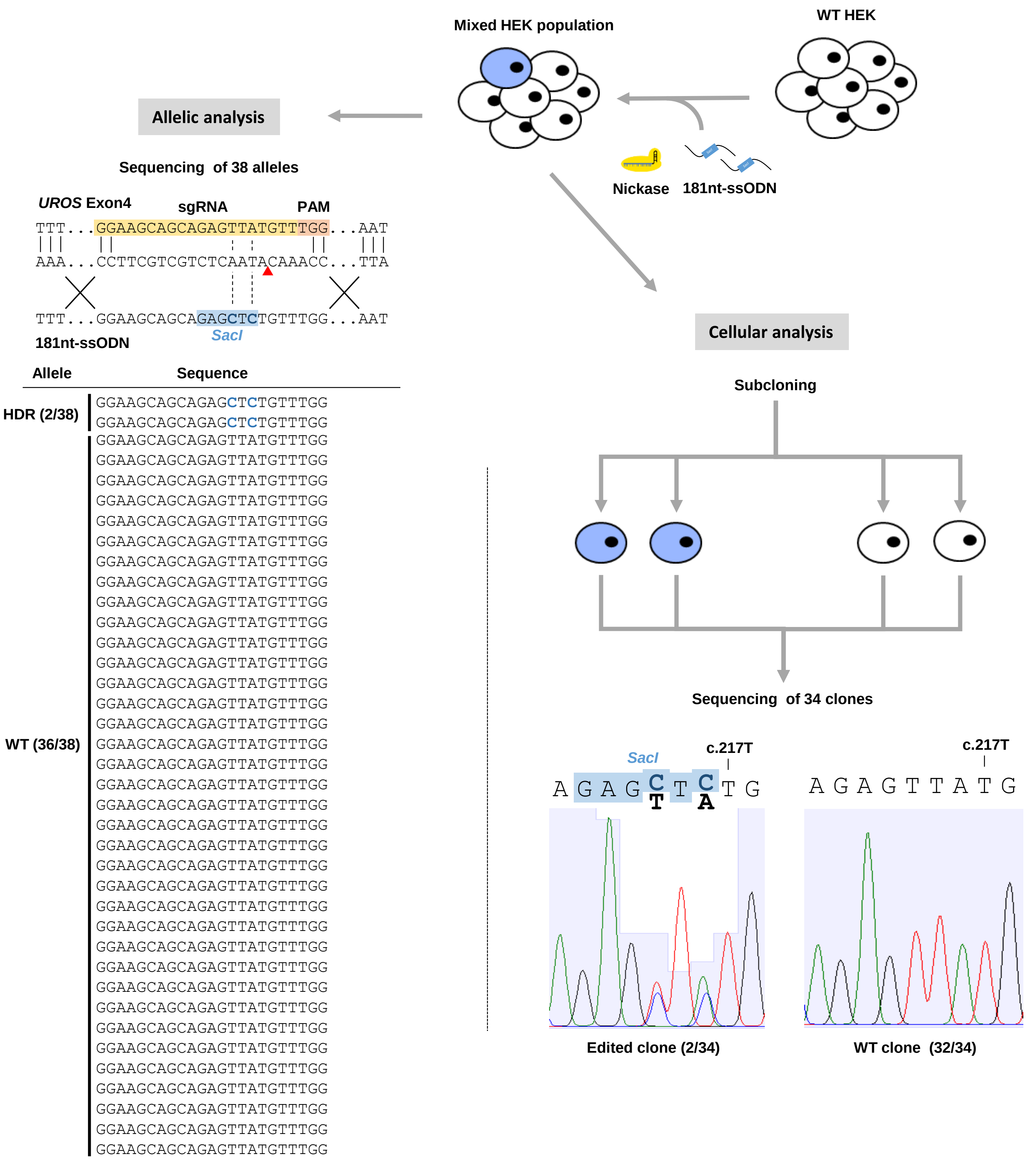

Supplementary Figure 5: Single nickase-mediated approach allows precise genome editing. Scheme for *UROS* exon 4 editing on WT HEK293T using nickase and a 181nt-ssODN. Transfected cells were then analyzed at allelic and cellular levels. (Left) For allelic analysis, 38 PCR products were isolated using TOPO vector and then sequenced. (Right) For cellular analysis, 34 transfected cells were subcloned and then sequenced. Frequencies and illustrative Sanger sequences of an edited clone and a WT clone. Cells are considered to be edited if they carry at least one *SacI*-edited allele and no indel-modified allele. HDR events are in blue.

Supplementary Figure 6

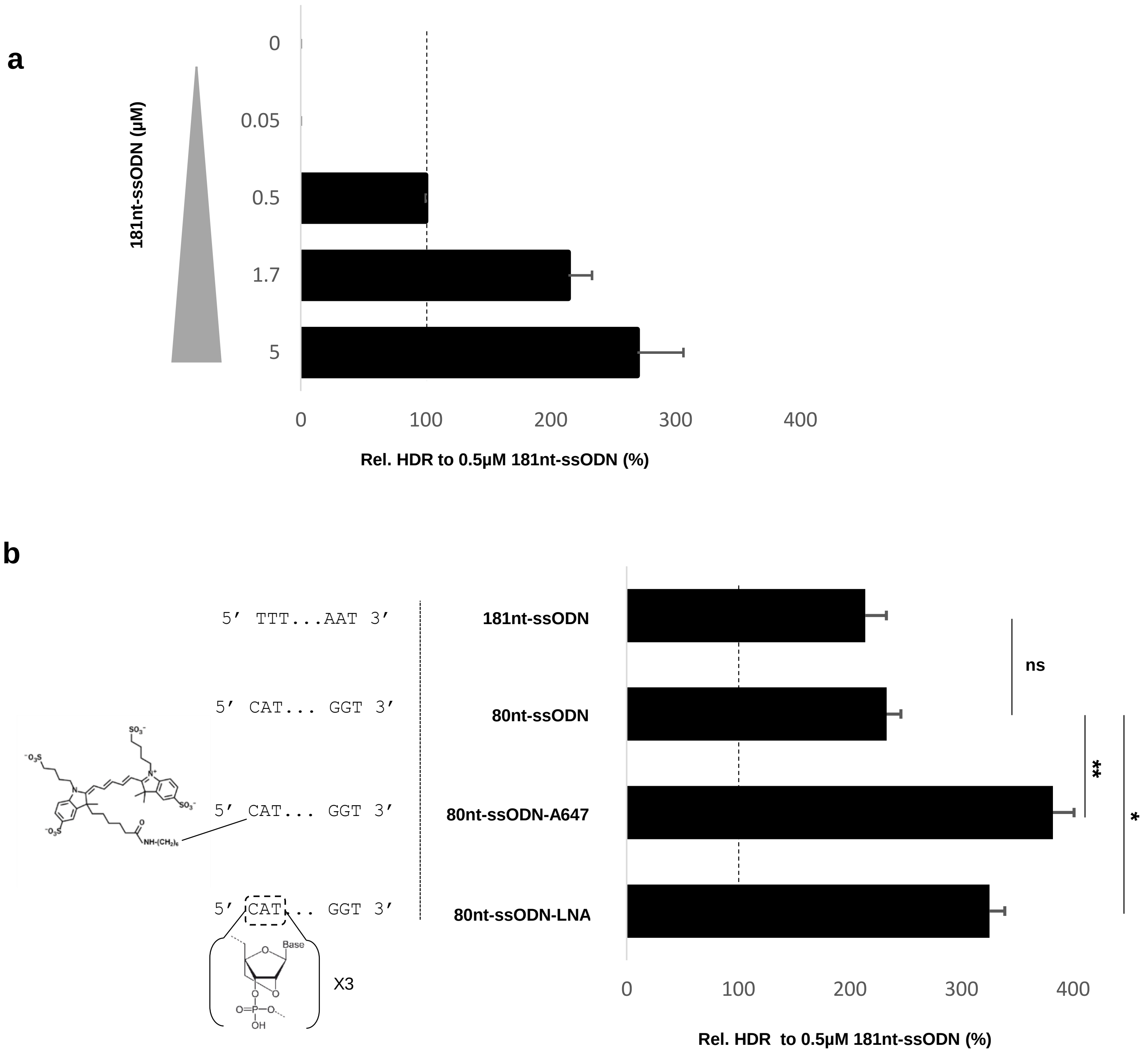

Supplementary Figure 6: Increased ssODN template concentration and 5' terminal modifications improve single nickase-mediated HDR efficiency. a, HDR frequencies for HEK293T cells transfected with nickase and co-delivered or not with 181nt-ssODN (0, 0.05, 0.5, 1.7, 5.1 µM) (n=5). **b**, HDR relative frequencies from cells transfected with nickase co-delivered either with 181nt-ssODN, 80nt-ssODN, 80nt-ssODN-A647 or 80nt-ssODN modified at its 5' terminal end with three Locked Nucleic Acid (80nt-ssODN-LNA) (n=5). Values are normalized with 181nt-ssODN condition at 0.5µM. Results are presented as mean ± SEM. Data are from 5 independent experiments. Statistical significance is inferred **a**, using paired one-way ANOVA, **b**, using two-tailed paired t-test to compare 80nt-ssODN vs 181nt-ssODN conditions and paired one-way ANOVA is used to compare 80nt-ssODN vs 80nt-ssODN-A647 and 80nt-ssODN-LNA. ns, not significant; *, $p<0.05$. For (a) and (b), source data are provided as a Source data file.

Supplementary Figure 7

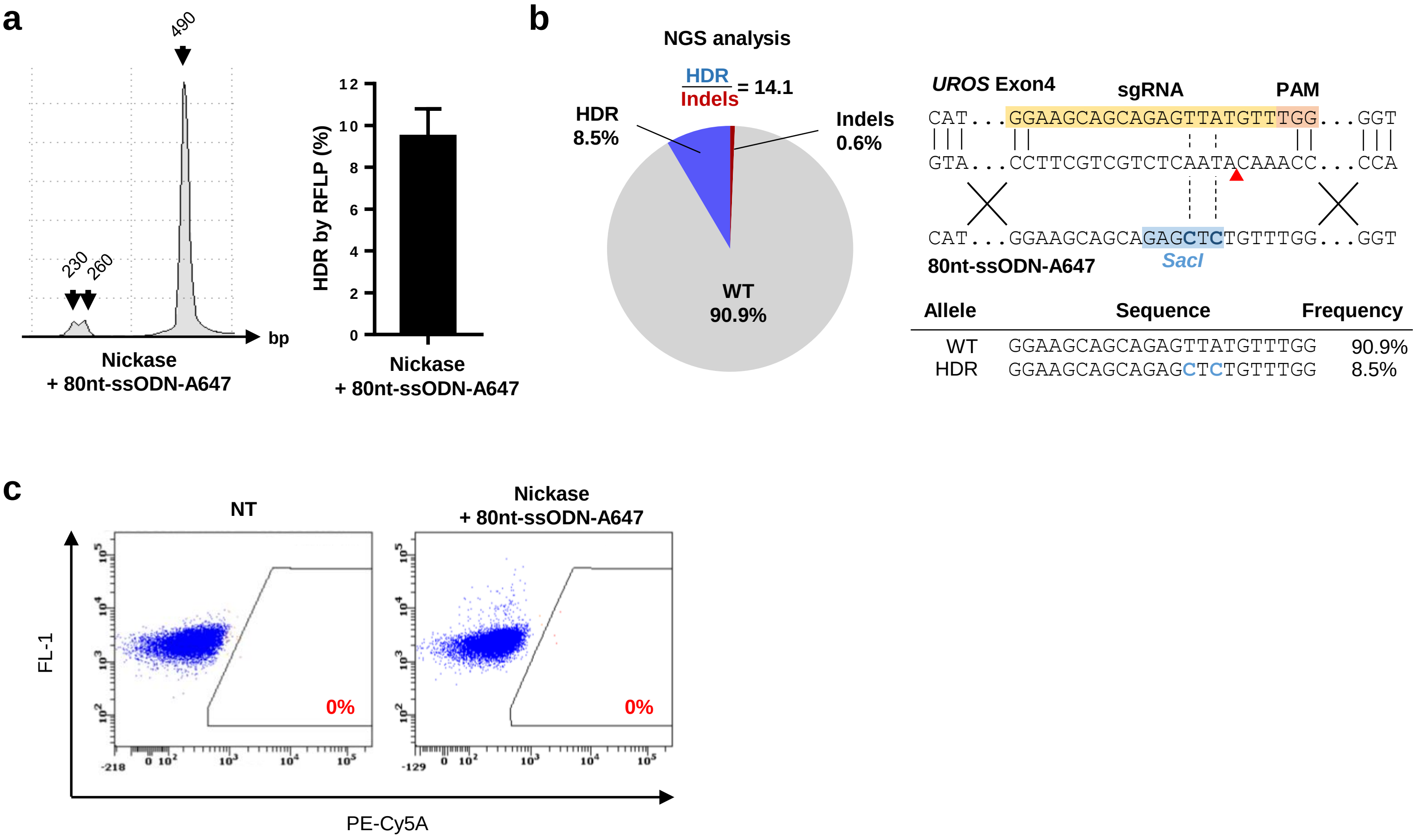

Supplementary Figure 7: Single nickase-mediated approach allows precise genome editing in K-562 cells. a, (Left) Illustrative RFLP analysis and (Right) HDR frequency for K-562 cells transfected with nickase and 80nt-ssODN-A647 (n=6). **b**, (Left) NGS analysis of allelic outcomes and associated HDR/indel ratio following transfection of K-562 cells with nickase and 80nt-ssODN-A647. (Right) Most common observed alleles (with frequencies ≥1%) aligned on the sgRNA sequence. HDR events in blue. **c**, Illustrative FACS results from non-transfected K-562 cells (NT) or K-562 transfected with nickase and 80nt-ssODN-A647. For (a), source data are provided as a Source data file.

Supplementary Figure 8

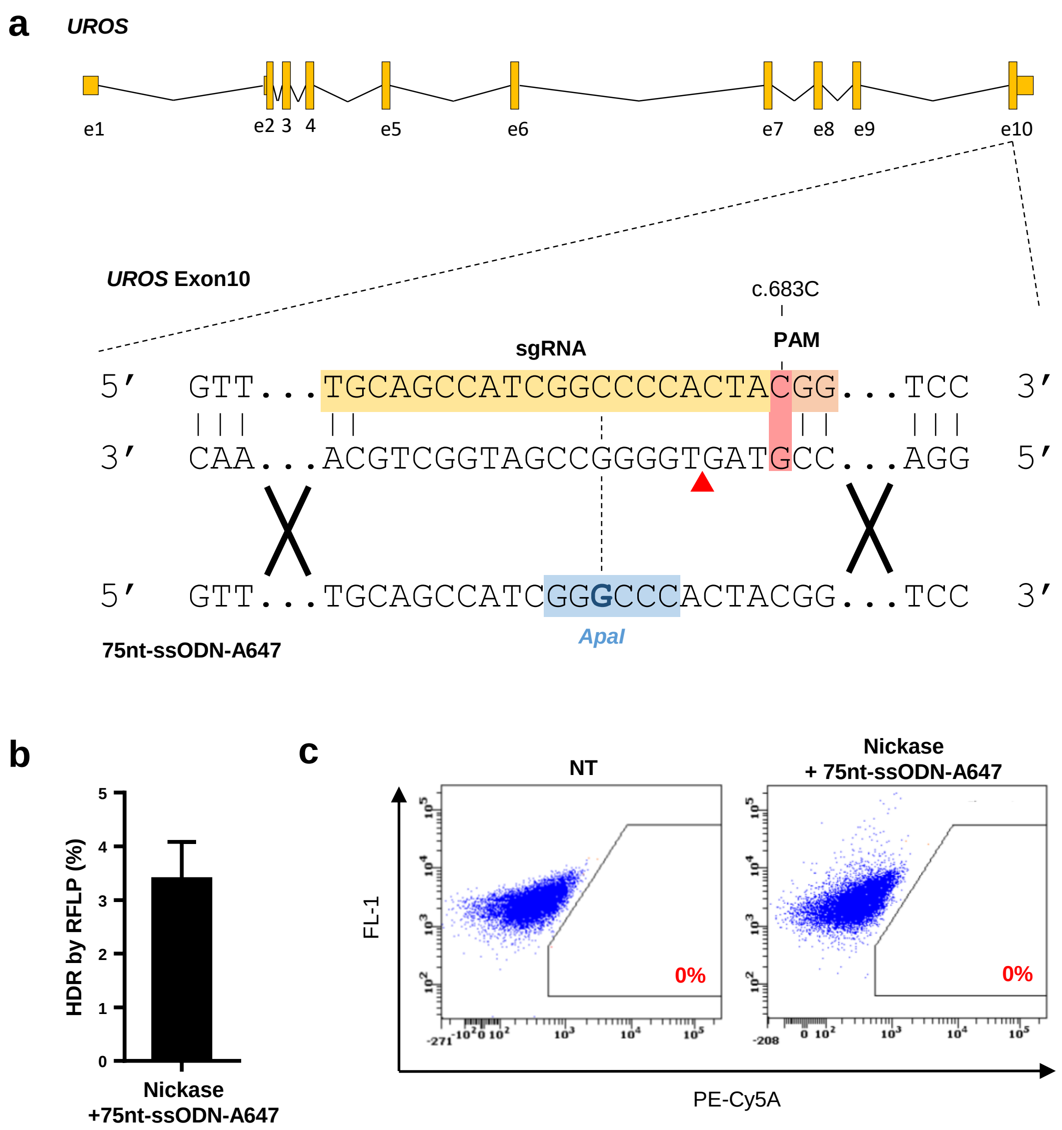

Supplementary Figure 8: Single nickase-mediated approach allows precise genome editing in *UROS* exon 10 locus. a, (Top) *UROS* gene overview and (Bottom) detailed view of exon 10 region and CRISPR-mediated HDR design using a c.683C-targeting sgRNA and a 75nt-ssODN-A647 carrying a silent *ApaI* restriction site (blue) close to c.683C position and in the sgRNA seed sequence. Expected cleavage position using a nickase is indicated with a red arrow. **b**, HDR frequency for transfected HEK293T cells with nickase and 75nt-ssODN-A647 (n=6). **c**, Illustrative flow cytometry results from non-transfected (NT) HEK293T cells or co-transfected with nickase and 75nt-ssODN-A647. For (b), source data are provided as a Source data file.

Supplementary Figure 9

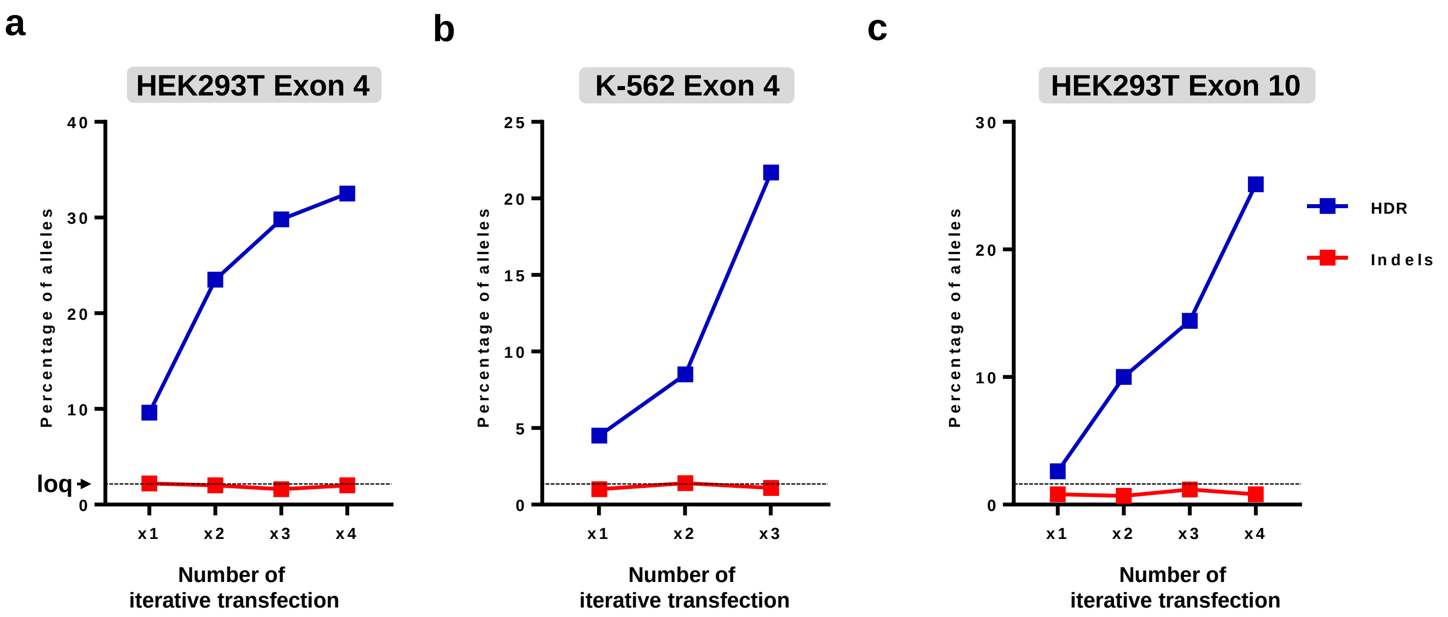

Supplementary Figure 9: Efficient HDR using iterative rounds of single nickase editing. HDR and indel quantification by TIDER analysis. Editing of **a,** *UROS* exon 4 in HEK293T; **b,** *UROS* exon 4 in K-562 cells and **c,** *UROS* exon 10 in HEK293T after 1 to 4 iterative transfections. HDR and indel rates are respectively presented in blue and red lines. loq: limit of quantification. For (a), (b) and (c), source data are provided as a Source data file.

Supplementary Figure 10

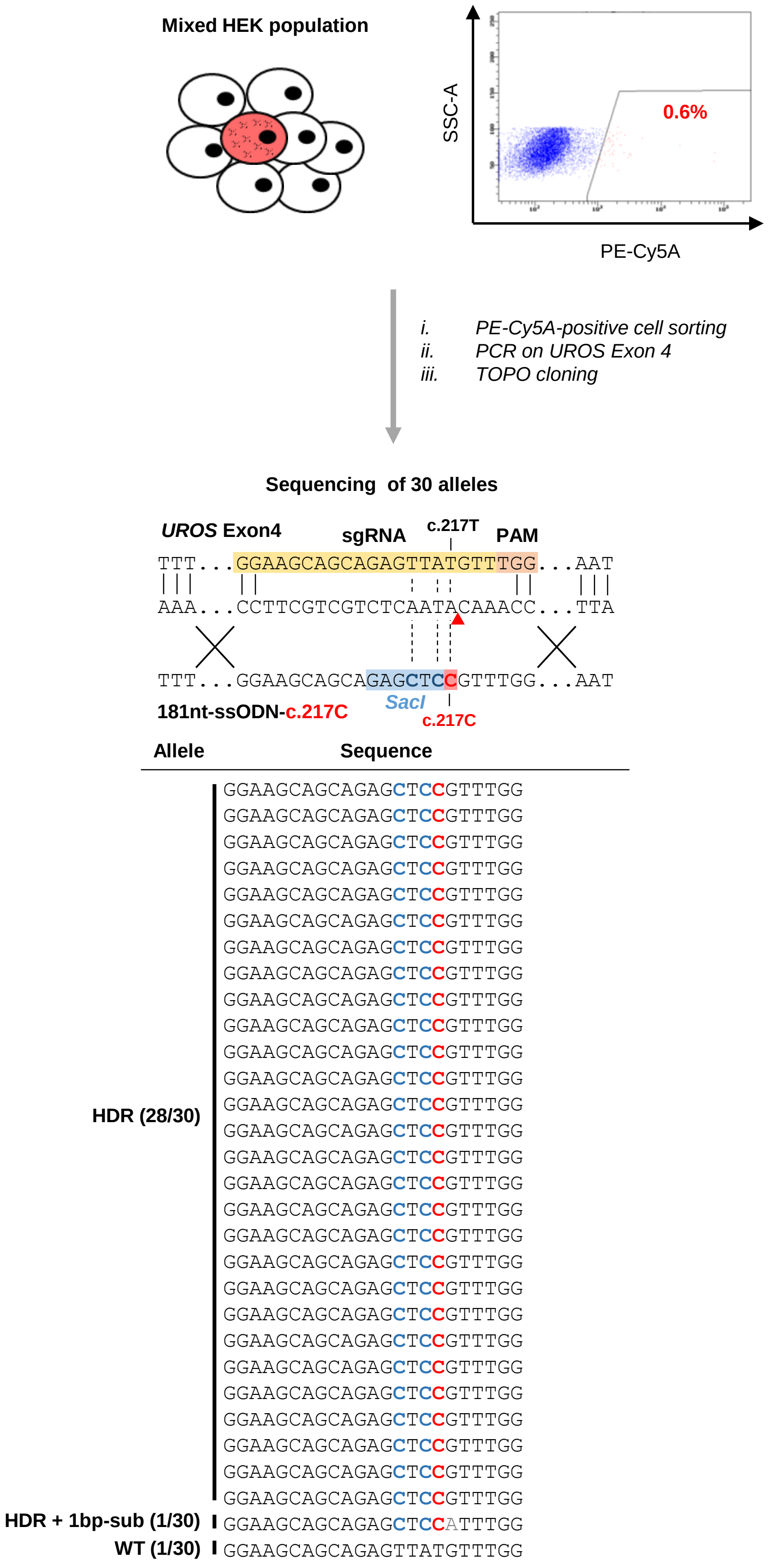

Supplementary Figure 10: Single nickase-mediated gene editing allows precise insertion of *SacI* and c.217C in fluorocytes during CEP modeling. Allelic analysis of PE-CyA5-positive sorted cells following transfection with nickase and a 181nt-ssODN-c.217C. PCR products were isolated using TOPO vector and then sequenced. HDR-mediated insertions of *SacI* and c.217C mutation are respectively in blue and red. Substitution in grey.

Supplementary Figure 11

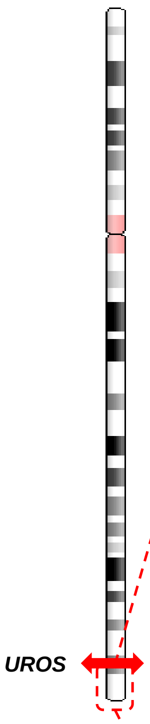

Region downstream *UROS* locus

Proto-oncogene
Tumor suppressor

UROS	-	Hydroxymethylbilane hydrolyase
BCCIP	-	BRCA2 and CDKN1A interacting protein
DHX32	-	DEAH-box helicase 32 (putative)
FANK1	-	Fibronectine type III and ankyrin repeat domains 1
ADAM12	-	ADAM metallopeptidase domain 12
C10orf90	-	Chromosome 10 open reading frame
FAM196A	-	Family with sequence similarity196 member A
DOCK1	-	Dedicator of cytokinesis 1
NPS	-	Neuropeptide S
FOXI2	-	Forkhead box I2
CLRN3	-	Clarin 3
PTPRE	-	Protein tyrosine phosphatase. receptor type E
MKI67	-	Marker of proliferation Ki-67
MGMT	-	6-0-Methylguanine-DNA-methyltransferase
EBF3	-	Early B cell factor 3
C10orf143	-	Chr10 open reading frame 143
GLRX3	-	Glutaredoxin 3
TCERG1L	-	Transcription elongation regulator 1 like
PPP2R2D	-	Protein phosphatase 2 regulatory subunit Bdelta
BNIP3	-	BCL2 interacting protein 3
JAKMIP3	-	Janus kinase and microtubule interacting protein 3
DPYSL4	-	Dihydropyriminidase like 4
STK32C	-	Serine/Threonine kinase 32C
PWWP2B	-	PWW domain containing 2B
INPP5A	-	OTTHUMP00000020775
NKX6-2	-	NK homeobox 2
CFAP46	-	Cilia and flagella-associated protein 46
ADGRA1	-	Adhesion G protein-coupled receptor A1
KNDC1	-	Kinase non catalytic C domain
UTF1	-	Undifferentiated embryonic cell transcription factor 1
VENTX	-	hCG_1747835
MIR202	-	microRNA 202
ADAM8	-	ADAM metallopeptidase domain 8
TUBGCP2	-	Tubulin gamme complex associated protein 2
CALY	-	Calcyon neuron specific vesicular protein
PRAP1	-	Proline rich acidic protein
FUOM	-	Fucose mutarotase
ECHS1	-	Enoyl-CoA hydratase. short-chain 1
MTG1	-	Mitochrondrial ribosome associated GTPase I
SPRN	-	Shadow of prion protein
CYP2E1	-	Cytochrome P450 family 2 subfamily E member 1
SYCE1	-	Synaptomenal complex central element protein
FRG2B	-	FSHD region gene 2 family member B

Genome Data Viewer (GRCh38.p12)

Supplementary Figure 11: List of genes downstream of *UROS* locus in Chr10. (Left) Schematic *UROS* locus position on chromosome 10 and (Right) list of 43 genes situated downstream. Genes known to be proto-oncogene or tumor-suppressor are respectively highlighted in red and blue.

Supplementary Table 1

Target	Target sequence	MIT Score	Number of mismatches	Indels (%)		Position (GRCh38)	Strand	Type	Gene
				Nuclease	Nickase				
On- target	GGAAGCAGCAGAGTTATGTTTGG	0	0	**83**	**<loq**	Chr10:125815054-125815077	+	Exonic	*UROS*
Off-target 1	GGCAGCAGCAGGGTTATGTTGGG	3.9	2	**5**	**<loq**	Chr16:2689598-2689620	+	Intergenic	-
Off-target 2	GGCAGCAGCAGGGTTATGTTGGG	3.9	2	**5**	**<loq**	Chr16:2609502- 2609524	+	Intergenic	-
Off-target 3	GAAAGCAGCAGAGTCATGTTGGG	3	2	**32**	**<loq**	Chr9:107494173- 107494195	-	Intergenic	-
Off-target 4	AGAAGCATCAGGGTTATGTTAGG	1.4	3	**<loq**	**<loq**	Chr4:103916635- 103916657	-	Intergenic	-
Off-target 5	TCAACCACCAGAGTTATGTTTGG	1.4	4	**<loq**	**<loq**	Chr21:15731392- 15731414	+	Intronic	*USP25*
Off-target 6	GGGAGCACCAGAGTGATGTTAGG	0.8	3	**<loq**	**<loq**	Chr1:16199632- 16199654	-	Intronic	*ARHGEF19*
Off-target 7	GTTAGGAGCTGAGTTATGTTGGG	0.7	4	**<loq**	**<loq**	Chr7:18471631- 18471653	-	Intronic	*HDAC9*
Off-target 8	GGCATTAGCTGAGTTATGTTAGG	0.7	4	**<loq**	**<loq**	Chr15:58511066- 58511088	-	Intergenic	-
Off-target 9	AAAAGCAGCCAAGTTATGTTTGG	0.7	4	**< loq**	**<loq**	Chr11:91048325- 91048347	-	Intergenic	-
Off-target 10	CCAATCAGCAGAGTTATGTAAGG	0.7	4	**<loq**	**<loq**	Chr6:137332792- 137332814	+	Intergenic	-

Supplementary Table 1: c217T-targeting gRNA off-targets TIDER analysis, using nuclease or single nickase in HEK293T cells. loq: limit of quantification.

Supplementary Table 2

**PCR primers sequence (5' to 3')**	
UROS Exon 4 Forward	TAGTTCCAGGCACATAGTAAGCAC
UROS Exon 4 Reverse	AGGAGGTGAACAACGAATAGACAG
NGS - UROS Exon 4 Forward	TCTTGTGGAAAGGACGAAACACCGGGGGACTCATTTTTACCAGCC
NGS - UROS Exon 4 Reverse	TCTACTATTCTTTCCCCTGCACTGTTCTGGAATTTAGTCTCCCAGCA
UROS Exon 10 Forward	GAGAGCGTGGATGCAGA
UROS Exon 10 Reverse	ACAGCAACCATACACTCAG
NGS - UROS Exon 10 Forward	TCTTGTGGAAAGGACGAAACACCGGTGTGCTGAAGCCCTGTTTT
NGS- UROS Exon 10 Reverse	TCTACTATTCTTTCCCCTGCACTGTGAGCCTTCCTGATGCCAGT
**sgRNA sequence (5' to 3')**	
Exon 4 sgRNA Forward	**GGAAGCAGCAGAGTTATGTT**
Exon 4 sgRNA Reverse	**ATAACTCTGCTGCTTCCGGT**
Exon 4 c.217C-SacI- specific sgRNA Forward	**GGAAGCAGCAGAGCTCCGTT**
Exon 4 c.217C-SacI- specific sgRNA Reverse	**AACGGAGCTCTGCTGCTTCC**
Exon 10 sgRNA Forward	**TGCAGCCATCGGCCCCACTA**
Exon 10 sgRNA Reverse	**TAGTGGGGCCGATGGCTGCA**
**HDR template sequence (5' to 3')**	
181nt-ssODN	TTTTGGTGTGCAGCTTTCTCATCCTGAAGATTACGGGGGACTCATTTTTACTAGCCCCAGAGCAGTGGAAGCAGCA**GAGCTCT**GTTTGGAGCAAAACAATAAAACTGAAGGTGAGGGTGGGTCTGCTGTCGATTCCACTGGACATTTATTTACTCTTATTTCTCCTGCTGGGAGACTAAAT
181nt-ssODN-c.217C	TTTTGGTGTGCAGCTTTCTCATCCTGAAGATTACGGGGGACTCATTTTTACTAGCCCCAGAGCAGTGGAAGCAGCA**GAGCTCC**GTTTGGAGCAAAACAATAAAACTGAAGGTGAGGGTGGGTCTGCTGTCGATTCCACTGGACATTTATTTACTCTTATTTCTCCTGCTGGGAGACTAAAT
80nt-ssODN	CATTTTTACCAGCCCCAGAGCAGTGGAAGCAGCA**GAGCTCT**GTTTGGAGCAAAACAATAAAACTGAAGGTGAGGGTGGG**T**
75nt-ssODN	GTTTTCTCTTCTGTCTTTATAGTTTGCAGCCATC**GGGCCC**ACTACGGCTCGCGCGCTGGCCGCCCAGGGCCTTCC
**PCR primers for off-target analysis (5' to 3' sequence)**	
Off-target 1 Forward	AGGTGAAGGTTTTGTGTGCGA
Off-target 1 Reverse	CAGTGAGGAATGAGGGTGGAG
Off-target 2 Forward	AGGTGAAGGTTTTGTGTGCGA
Off-target 2 Reverse	CAGTGAGGAATGAGGGTGGAG
Off-target 3 Forward	ACCCTCTGTCTTGTGTAGACT
Off-target 3 Reverse	AGCCTCAGCCTCATCATGTTT
Off-target 4 Forward	ACTGTCCACCATTTCTCACCA
Off-target 4 Reverse	ATCCTTCTCTTTGCCCAGACC
Off-target 5 Forward	GGGCAAAAGAGTAAACCGTGC
Off-target 5 Reverse	CCATGTGCTCTTTCCTAAGCC
Off-target 6 Forward	CCACGTTCAAGCAGGCATAC
Off-target 6 Reverse	GGGTGGGGGTAAGCTCTCTA
Off-target 7 Forward	ATGGGTTAGGTGAAAGCTCAGT
Off-target 7 Reverse	ACTAGTTGCATCTTCCTAGCATC
Off-target 8 Forward	TCAACAACCGCACCTGGAAG
Off-target 8 Reverse	AACTTGCCTGGGCCCTAAC
Off-target 9 Forward	GAATATCTTACTCCCAGCGT
Off-target 9 Reverse	CATCATTCAGCTTAATTCACTTTGT

Supplementary Table 2: Oligonucleotide sequences used for PCR, sgRNA sequence and HDR template.
