# Supplementary material for: CRISPR-Cas9 genome editing induces megabase-scale chromosomal truncations
Source: Nat Commun. 2019 Mar 8;10:1136. doi: 10.1038/s41467-019-09006-2 (PMC6408493; doi:10.1038/s41467-019-09006-2)
Supplement: Supplementary file 2 — Reporting Summary [file 41467_2019_9006_MOESM2_ESM.pdf]

nature research

Corresponding author(s): François Moreau-Gaudry

Last updated by author(s): 31/1/2019

Reporting Summary

Nature Research wishes to improve the reproducibility of the work that we publish. This form provides structure for consistency and transparency in reporting. For further information on Nature Research policies, see Authors & Referees and the Editorial Policy Checklist.

Statistics

For all statistical analyses, confirm that the following items are present in the figure legend, table legend, main text, or Methods section.

n/a	Confirmed	
☐	☒	The exact sample size (*n*) for each experimental group/condition, given as a discrete number and unit of measurement
☐	☒	A statement on whether measurements were taken from distinct samples or whether the same sample was measured repeatedly
☐	☒	The statistical test(s) used AND whether they are one- or two-sided<br>*Only common tests should be described solely by name; describe more complex techniques in the Methods section.*
☒	☐	A description of all covariates tested
☒	☐	A description of any assumptions or corrections, such as tests of normality and adjustment for multiple comparisons
☐	☒	A full description of the statistical parameters including central tendency (e.g. means) or other basic estimates (e.g. regression coefficient) AND variation (e.g. standard deviation) or associated estimates of uncertainty (e.g. confidence intervals)
☐	☒	For null hypothesis testing, the test statistic (e.g. *F*, *t*, *r*) with confidence intervals, effect sizes, degrees of freedom and *P* value noted<br>*Give P values as exact values whenever suitable.*
☒	☐	For Bayesian analysis, information on the choice of priors and Markov chain Monte Carlo settings
☒	☐	For hierarchical and complex designs, identification of the appropriate level for tests and full reporting of outcomes
☒	☐	Estimates of effect sizes (e.g. Cohen's *d*, Pearson's *r*), indicating how they were calculated

Our web collection on statistics for biologists contains articles on many of the points above.

Software and code

Policy information about availability of computer code

Data collection	n/a
Data analysis	Graph Pad Prism 6 software, Tracking of Insertions, DEletions and Recombination events (TIDER) and CHOPCHOP v2 algorithm, Diva 2BD

For manuscripts utilizing custom algorithms or software that are central to the research but not yet described in published literature, software must be made available to editors/reviewers. We strongly encourage code deposition in a community repository (e.g. GitHub). See the Nature Research guidelines for submitting code & software for further information.

Data

Policy information about availability of data

All manuscripts must include a data availability statement. This statement should provide the following information, where applicable:

- Accession codes, unique identifiers, or web links for publicly available datasets
- A list of figures that have associated raw data
- A description of any restrictions on data availability

All data generated or analysed during this study are included in this published article (and its supplementary information files).

Field-specific reporting

Please select the one below that is the best fit for your research. If you are not sure, read the appropriate sections before making your selection.

☒ Life sciences ☐ Behavioural & social sciences ☐ Ecological, evolutionary & environmental sciences

For a reference copy of the document with all sections, see nature.com/documents/nr-reporting-summary-flat.pdf

Life sciences study design

All studies must disclose on these points even when the disclosure is negative.

Sample size	n/a
Data exclusions	no data exclusion
Replication	At least 3 independent experiments
Randomization	n/a
Blinding	n/a

Reporting for specific materials, systems and methods

We require information from authors about some types of materials, experimental systems and methods used in many studies. Here, indicate whether each material, system or method listed is relevant to your study. If you are not sure if a list item applies to your research, read the appropriate section before selecting a response.

Materials & experimental systems

n/a	Involved in the study	
☐	☐	Antibodies
☐	☒	Eukaryotic cell lines
☐	☐	Palaeontology
☐	☐	Animals and other organisms
☐	☐	Human research participants
☐	☐	Clinical data

Methods

n/a	Involved in the study	
☐	☐	ChIP-seq
☐	☒	Flow cytometry
☐	☐	MRI-based neuroimaging

Antibodies

Antibodies used	n/a
Validation	n/a

Eukaryotic cell lines

Policy information about cell lines

Cell line source(s)	ATCC for HEK293T, K562 and fibroblasts (CRL 4001, BJ-5ta)
Authentication	Authenticated by ATCC
Mycoplasma contamination	PCR-free mycoplasma
Commonly misidentified lines (See ICLAC register)	n/a

Palaeontology

Specimen provenance	n/a
Specimen deposition	n/a
Dating methods	n/a

☒ Tick this box to confirm that the raw and calibrated dates are available in the paper or in Supplementary Information.

Animals and other organisms

Policy information about studies involving animals; ARRIVE guidelines recommended for reporting animal research

Laboratory animals	the study did not involved laboratory animal
Wild animals	the study did not involved wild animal
Field-collected samples	the study did not involve samples collected from the field
Ethics oversight	Human foreskin fibroblasts (HFF) were from a healthy person in accordance with the ethical standards of the committee responsible for human experimentation (Centre Hospitalier Universitaire de Bordeaux).

Note that full information on the approval of the study protocol must also be provided in the manuscript.

Human research participants

Policy information about studies involving human research participants

Population characteristics	n/a
Recruitment	n/a
Ethics oversight	n/a

Note that full information on the approval of the study protocol must also be provided in the manuscript.

Clinical data

Policy information about clinical studies

All manuscripts should comply with the ICMJE guidelines for publication of clinical research and a completed CONSORT checklist must be included with all submissions.

Clinical trial registration	n/a
Study protocol	n/a
Data collection	n/a
Outcomes	n/a

ChIP-seq

Data deposition

- [ ] Confirm that both raw and final processed data have been deposited in a public database such as GEO.
- [ ] Confirm that you have deposited or provided access to graph files (e.g. BED files) for the called peaks.

Data access links<br>*May remain private before publication.*	n/a
Files in database submission	n/a
Genome browser session<br>(e.g. UCSC)	n/a

Methodology

Replicates	n/a
Sequencing depth	n/a
Antibodies	n/a
Peak calling parameters	n/a
Data quality	n/a
Software	n/a

Flow Cytometry

Plots

Confirm that:

☒ The axis labels state the marker and fluorochrome used (e.g. CD4-FITC).

☒ The axis scales are clearly visible. Include numbers along axes only for bottom left plot of group (a 'group' is an analysis of identical markers).

☒ All plots are contour plots with outliers or pseudocolor plots.

☒ A numerical value for number of cells or percentage (with statistics) is provided.

Methodology

Sample preparation	After trypsinization, cells were washed in PBS and resuspended in PBS-EDTA. No antibodies are necessary to observe fluorocytes.
Instrument	FACSCanto, BD, Franklin Lakes, NJ, USA Cells were sorted by BD FACS Aria ®
Software	Diva 2BD
Cell population abundance	After cell sorting purity is evaluated (need to be > 95%)
Gating strategy	FSC/SSC gate allows elimination of dead cells before FACS analysis. A positive population is quantified and isolated only if a valley is present between the positive and negative population

☐ Tick this box to confirm that a figure exemplifying the gating strategy is provided in the Supplementary Information.

Magnetic resonance imaging

Experimental design

Design type	n/a
Design specifications	n/a
Behavioral performance measures	n/a

Acquisition

Imaging type(s)	n/a
Field strength	n/a
Sequence & imaging parameters	n/a
Area of acquisition	n/a

Diffusion MRI ☐ Used ☒ Not used

Preprocessing

Preprocessing software	n/a
Normalization	n/a
Normalization template	n/a
Noise and artifact removal	n/a
Volume censoring	n/a

Statistical modeling & inference

Model type and settings	n/a
Effect(s) tested	n/a

Specify type of analysis: ☐ Whole brain ☐ ROI-based ☐ Both

Statistic type for inference<br>(See Eklund et al. 2016)	n/a
Correction	n/a

Models & analysis

n/a	Involved in the study
☒	☐ Functional and/or effective connectivity
☒	☐ Graph analysis
☒	☐ Multivariate modeling or predictive analysis
